# Anti-Diabetic Obesity Effects of *Wasabia Japonica* Matsum Leaf Extract on 45% Kcal High-Fat Diet-Fed Mice

**DOI:** 10.3390/nu12092837

**Published:** 2020-09-16

**Authors:** Beom-Rak Choi, Hyun-Jee Kim, Young-Joon Lee, Sae-Kwang Ku

**Affiliations:** 1 Research Institute, Nutracore Co., Ltd., Gwanggyo SK Viewlake A-3206, Beobjo-Ro 25, Yeongtong-Gu, Suwon, Gyeonggi-Do 16514, Korea; brchoi@nutracore.co.kr; 2 Department of Anesthesiology and Pain Medicine, School of Medicine, Kyungpook National University, 130 Dongdeok-ro, Jung-gu, Daegu 41944, Korea; hj_kim@knu.ac.kr; 3 Department of Preventive Medicine, College of Korean Medicine, Deagu Haany University, 1, Haanydaero, Gyeongsan, Gyeongsangbuk-Do 38610, Korea; 4 Department of Histology and Anatomy, College of Korean Medicine, Daegu Haany University, 1, Haanydaero, Gyeongsan, Gyeongsangbuk-Do 38610, Korea

**Keywords:** wasabi leaf/folium, *Wasabia japonica* (miq.) matsum, obesity, diabetes, hepatic glucose-regulating enzyme, lipid metabolism-related gene expression

## Abstract

The present study examined the effects of Wasabi leaf (WL) on 45% Kcal high-fat diet (HFD)-fed mild diabetic obese mice. In particular, the hepatoprotective (i.e., liver weight, histopathology of liver, serum aspartate aminotransferase, alanine aminotransferase, and gamma-glutamyltransferase) effects of 12 weeks of continuous oral administration of 250 mg/kg metformin, and 200, 100, or 50 mg/kg WL were investigated. In addition, the hypolipidemic (i.e., serum triglyceride, total cholesterol, high-density lipoprotein-cholesterol, and low-density lipoprotein levels), hypoglycemic (i.e., glycated hemoglobin, blood glucose and insulin levels, pancreatic weight, and immunohistochemical-histopathological analysis of the pancreas), and anti-obesity effects (i.e., body weight, mean food consumption, total and abdominal body fat mass, periovarian fat weight, and histopathology of the periovarian and abdominal wall adipocytes) were monitored. The liver and general antioxidant defense systems were also assessed by lipid metabolism-related gene expression. All diabetes manifestations and related complications, including obesity and non-alcoholic fatty liver disease (NAFLD), were dose-dependently reduced after 84 days of oral treatment with metformin or each of the three dosages of WL. In particular, 50 mg/kg WL showed effective suppression effects against HFD-induced diabetes and related complications of obesity, NAFLD, and hyperlipidemia, comparable to the effects of metformin.

## 1. Introduction

The increasing incidence of obesity worldwide is highly correlated with a metabolic syndrome known as type II diabetes, which results from high-calorie intake and physical inactivity [1]. The number of individuals with this syndrome is expected to double to more than 300 million by 2025 [2]. A critical determinant for obesity may be an augmentation in the regional fat distribution, particularly abdominal obesity. Notably, abdominal obesity is often clustered with atherogenic risk factors [3], such as hypertension, dyslipidemia, changes in coagulation and inflammatory cytokine profiles, and insulin resistance. Consequences include increased mortality and morbidity from cardiovascular disease [4]. Excessive consumption of fatty acids induced the accumulation of triglycerides (TGs) in fat tissue, where lipolysis increases. High levels of fatty acids, related with elevated lipolysis in insulin-resistant adipocytes, consequently lead to an excess of fatty acids in non-adipose tissues (e.g., muscle, liver, and pancreas). Augmented levels of tissue fatty acid transport and binding proteins in non-adipose and adipose tissues facilitate uptake in insulin-resistant individuals. The enhanced utilization of free fatty acids and deposition in muscle makes a negative feedback loop involving insulin-mediated muscle insulin signaling and glucose availability. Prolonged exposure of the pancreas to free fatty acids impairs insulin release through a lipotoxic mechanism [5]. High free fatty acid concentrations in the liver cause insulin resistance through enhanced glucose output by the liver [6]. The increase of TGs in the liver caused by high concentrations of free fatty acids results in non-alcoholic fatty liver disease (NAFLD). Notably, NAFLD damages the liver, causing steatohepatitis, steatosis, and hepatocellular necrosis to fibrosis [7]. The equilibrium between fat synthesis and fat breakdown is pivotal for amelioration of insulin resistance and NAFLD. These characteristics appear commonly in metabolic syndrome [8].

No pharmacological approach has been approved for treatment of metabolic syndrome [9]. Several drug treatments have been suggested, but none of these drugs have remarkable efficacy for the entire spectrum of metabolic syndrome [10]; they also exhibit multiple restrictions, such as high rates of secondary failure and various adverse effects [11]. Lifestyle interventions, including exercise and weight loss, are the only established treatments for metabolic syndrome; however, these interventions are frequently hard for individuals with metabolic syndrome to maintain for extended periods [9]. Thus, patients with metabolic syndrome and experts are contemplating complementary and alternative methods [12,13,14]. There is a need to discover agents targeted at elevated hepatic lipids, which are safe for long-term administration. As a result that suppression of oxidative stress and regulation of postprandial hyperglycemia are pivotal to the management of diabetes [15,16], many attempts have been made to find effective antioxidants and α-glucosidase inhibitors from natural materials to develop physiologically functional foods or lead compounds to cure diabetes [16,17,18]. Among these candidate compounds, natural extracts (including wasabi leaf (WL)) have antioxidant, anti-inflammatory, and anti-obesity effects [19,20].

Wasabi (*Wasabia/Eutrema japonica* Matsum) is a native Japanese plant that was used medicinally in ancient times [21]. As wasabi show strong pungent characteristics, including isothiocyanates such as allyl isothiocyanate, it is used as a spice or pickling agent [21]. Most of the reported studies have focused the antimicrobial [22], antioxidative [23], and anti-carcinogenic activities [24] of isothiocyanate. However, there are few studies investigating the functional properties of other components in wasabi, particularly in its summer leaves, which are considered late-growth with minimal pungency [25], and anti-obesity effects of wasabi leaf in mice [21] and rats [20] fed a high-fat diet (HFD) have been reported with in vitro assay [19]. In this study, we investigated the efficacy of WL in 45% Kcal high-fat diet (HFD)-fed mice, compared with metformin treatment, a representative anti-diabetic and anti-obesity drug used to treat type II diabetes. Furthermore, we carried out our experiments on HFD female mice. Difference in the prevalence of overweight and obesity among both men and women is affected through complex sociocultural pathways. There are gender differences in food consumption; women may eat more foods with sugar than men. Overall, the prevalence of obesity is higher in women than in men [26].

## 2. Materials and Methods

### 2.1. Animals and HFD

Forty-eight female SPF/VAF CrljOri:CD1[ICR] mice (OrientBio, Seungnam, Korea) were used after 10 days of acclimatization. The animals were assigned four or five per polycarbonate cage in a temperature- and humidity-controlled room (20–25 °C; 40–45%). The light:dark cycle was 12 h:12 h; standard rodent diet (Purinafeed, Seungnam, Korea) and water were free to access. Animals were supplied the 45% Kcal HFD (Research Diet, New Brunswick, NJ, USA) after acclimatization. The intact control mice were fed a normal fat diet (NFD; Purinafeed, Seungnam, Korea) instead of the HFD. Animals that adapted to the HFD were selected and allocated to one of five groups (Eight mice per group, total 40 HFD supplied mice, and 8 NFD supplied mice) according to the body weight. All laboratory animals were treated in accordance with the national regulations regarding the use and welfare of laboratory animals; experimental protocols were approved by the Institutional Animal Care and Use Committee of Daegu Haany University (Gyeongsan, Gyeongbuk, Korea) before the experiment began (Approval No. DHU2019-088).

### 2.2. Preparation and Administration of the Test Substances

WL was prepared and supplied by NUTRACORE (Suwon, Korea). Metformin hydrochloride (Wako, Osaka, Japan) was used as the reference recommended drug. In this study, 200 mg/kg was selected as the highest WL dose according to the previous study [27]; the middle and lowest WL doses were 100 and 50 mg/kg using common ratio 2. The administration volume was 10 mL/kg, which is the general dosing volume for mice [28]. The dose level of metformin was 250 mg/kg; this was chosen based on the results in our previous animal studies [13,16,29,30]. WL was dissolved in distilled water as concentration of 20, 10, and 5 mg/mL, and administered through the oral route once per day for 84 days using a stainless-steel gastric gavage device attached to syringe, beginning 1 week after the HFD was offered. Metformin was dissolved in distilled water, which was used in the intact and HFD vehicle control mice for oral-administration.

### 2.3. Body Weight Changes, Mean Daily Food Consumption, and Body Fat Density (%)

Body weights were recorded 8 days (at just before beginning of HFD supply) and 1 day before start of WL treatment, as well as on the first administration day and weekly thereafter, by an electronic balance (XB320M, Precisa Instrument, Zuerich, Switzland). At the start of administration and at termination, all mice were fasted overnight to reduce differences from feeding. We measured bodyweight gains during the adaptation and administration periods. The amount of the diet remaining was measured 24 h after feeding by an electronic balance (XB320M, Precisa Instrument, Zuerich, Switzland) and divided by the number of animals bred in the cage to determine mean daily food consumption of each animal (g/day/mice). These evaluations were performed once per week during 84-day administration. Fat densities of the entire body and the abdominal cavity region of each mouse were detected by dual-energy X-ray absorptiometry (DEXA; InAlyzer; Medikors, Seungnam, Korea) at the end of the treatment of the test materials [30,31].

### 2.4. Blood Glucose Levels

After 84 days administration, blood was prepared from the vena cava under 2–3% isoflurane (Hana Pharm. Co., Hwasung, Korea) inhalation anesthesia in a mixture of 70% N_2_O and 28.5% O_2_, using an inhalation anesthesia apparatus (Surgivet, Waukesha, WI, USA) and ventilator (Model 687, Harvard Apparatus, Cambridge, UK). Plasma was separated in the sodium fluoride glucose vacuum tubes (Becton Dickinson, Franklin Lakes, NJ, USA). Levels of blood glucose were determined by an automated blood analyzer (Dri-Chem NX500i; Fuji Medical System Co., Ltd., Tokyo, Japan).

### 2.5. Serum Biochemistry, Insulin, and Blood Glycated Hemoglobin (HbA1c) Levels

Some blood collected from the vena cava was added to clotting activated serum tubes and centrifugation was performed at 15,000 rpm for 10 min to separate the serum. Alanine aminotransferase (ALT), aspartate aminotransferase (AST), lactate dehydrogenase (LDH), alkaline phosphatase (ALP), gamma-glutamyltransferase (GGT), TGs, total cholesterol, low-density lipoprotein (LDL), and high-density lipoprotein-cholesterol (HDL-C) contents were measured by an automated blood analyzer (Dri-Chem NX500i; Fuji Medical System Co., Ltd., Tokyo, Japan). Furthermore, blood hemoglobin A1c (HbA1c) and serum insulin contents were determined by automated HbA1c measurement equipment (Model Easya1c; Infopia, Anyang, Korea) and a mouse insulin enzyme-linked immunosorbent assay kit (Alpco Diagnostics, Windham, NH, USA).

### 2.6. Organ Weights

After sacrifice, the liver, pancreas, and abdominal wall deposited fat pad attached to the muscularis quadratus lumborum were weighed at the time of sacrifice. Relative weights were calculated by body weight at the time of sacrifice and absolute weight to reduce variations from individual body weights (% of body weights).

### 2.7. Fecal Lipid Composition

The extraction of lipids from feces were gathered 8 h following the last administration of last test substance [32]. The levels of fecal total cholesterol and TGs were determined using commercial kits (total cholesterol colorimetric assay kit, Cayman, Ann Arbor, MI, USA; Total Cholesterol Assay Kit (Colorimetric), Cell Biolabs, San Diego, CA, USA) and an absorbance microplate reader (Sunrise, Tecan, Männedorf, Switzerland). Briefly, the fecal samples were dried in dry oven. For 100 mg of sample, extract with 2 mL of a mixture of chloroform, isopropanol, and NP-40 was added in a homogenizer. The extract was centrifuged at 15,000× *g* for 10 min. The supernatant liquid was transferred to a new tube, and then dried at 50 °C. The samples were placed under vacuum condition for 30 min to remove the organic solvent. The dried lipids were dissolved in 200 μL of assay diluent. Then, the concentration of cholesterol in fecal samples was measured using a microplate reader (Sunrise, Tecan, Männedorf, Switzerland), according to the manufacture’s instruction [16,30,33].

### 2.8. Liver Antioxidant Defense Systems and Lipid Peroxidation

Hepatic tissues samples were homogenized using ultrasonic cell disruptor (KS-750, Madell Technology Corp., Ontario, CA, USA). Subsequently, the samples were centrifuged at 12,000× *g* for 15 min [34]. The level concentration of liver lipid peroxidation was measured by the thiobarbituric acid assay. A 0.1 mL of 25% trichloroacetic (Merck, West Point, CA, USA) acid was added to prepared hepatic homogenates and then centrifuged (1700× *g*, 40 min, 4 °C). Glutathione (GSH) level was measured at 412 nm with 2-nitrobenzoic acid (Sigma-Aldrich, St. Louise, MO, USA). Destruction of H_2_O_2_ in the existence of catalase (CAT) was determined spectrophotometrically at 240 nm. The activity of CAT was measured as the amount of enzyme that disintegrate 1 nM of H_2_O_2_ per minute at pH 7.8 and 25 °C. Due to the generation of superoxide radicals made by xanthine and xanthine oxidase, the activity of superoxide dismutase (SOD) was measured, which interact with nitrotetrazolium blue to form formazan dye. The activity of SOD was determined at 560 nm by the gradation of inhibition of this interaction. One unit of SOD enzyme activity is equivalent to the amount of enzyme that decreases the first absorbance of nitroblue tetrazolium by half in 1 min.

### 2.9. Glucose-Regulating Enzyme Activities in Liver

A hepatic tissue was homogenized in 0.1 M triethanolamine, 0.2 M EDTA, and 0.002 M dithiothreitol buffer solution and centrifuged at 4 °C for 15 min at 1000× *g*. The supernatant was centrifuged at 4 °C for 15 min at 10,000× *g*. The activity of glucokinase (GK) was measured. A 0.98-mL aliquot of the reaction mixture (50 mM Hepes-NaGT (pH 7.4), 100 mM KCl, 7.5 mM MgCl_2_, 2.5 mM dithioerythritol, 10 mg/mL albumin, 10 mM glucose, 4 units of glucose-6-phosphate dehydrogenase, 50 mM NAD+, and 10 μL hepatic tissue homogenates) was pre-incubated at 37 °C for 10 min. The reaction was started by mixing 10 μL of 5 mM ATP; and then, the mixed sample was incubated at 37 °C for 10 min. The alteration was measured at 340 nm absorbance. The activity of glucose-6-phosphatase (G6pase) was recorded. The reaction mixture was used containing 765 μL of 131.58 mM Hepes-NaGT, 100 μL of 18 mM EDTA, 100 μL of 265 mM glucose-6-phosphate, 10 μL of 0.2 M NADP+, 0.6 IU/mL mutarotase, and 0.6 IU/mL glucose dehydrogenase. Following the incubation performed at 37 °C for 3 min, 5 μL hepatic tissue homogenate was mixed with the reaction mixture. After incubation at 37 °C for 4 min, the change was recorded at 340 nm absorbance. The activity assay of phosphoenolpyruvate carboxykinase (PEPCK) was performed. The reaction mixture was used containing 72.92 mM sodium HEPES (pH 7.0), 10 mM dithiothreitol, 500 mM NaHCO3, 10 mM MnCl2, 25 mM NADH, 100 mM IDP, 200 mM PEP, 7.2 unit of malic dehydrogenase, and 10 μL of hepatic tissue homogenate. The activity of enzyme was calculated according to the reduction of the mixture at 340 nm absorbance and 25 °C, by a spectrophotometer (OPTIZEN POP, Mecasys, Daejeon, Korea). All reagents and chemicals from Sigma-Aldrich (St. Louis, MO, USA) were used for the measurement of the hepatic enzyme activity.

### 2.10. Analysis of Real-Time Reverse Transcription-Polymerase Chain Reaction (RT-PCR)

Acetyl-CoA carboxylase 1 (ACC1), AMP activated protein kinase (AMPK)α1, and AMPKα2 mRNA expression levels were determined in prepared hepatic tissues by real-time RT-PCR. In addition, peroxisome proliferator-activated receptor (PPAR)α, PPARγ, leptin, uncoupling protein (UCP)2, adiponectin, CCAAT-enhancer-binding protein (C/EBP)α, C/EBPβ, fatty acid synthase, and sterol-regulatory-element-binding protein 1c (SREBP1c) mRNA expression levels were determined separately in periovarian adipose tissues, using methods described in previous reports [35,36]. Briefly, RNA was extracted by TRIzol reagent (Invitrogen, Carlsbad, CA, USA). The RNA quality and concentrations were measured by the CFX96^TM^ Real-Time System (Bio-Rad, Hercules, CA, USA). Samples were mixed with recombinant DNase I (DNA-free; Ambion, Austin, TX, USA) to eliminate contaminating DNA. RNA was reverse transcribed by a reagent in the High-Capacity cDNA Reverse Transcription Kit, in accordance with the manufacturer’s instructions. ABI Step One Plus Sequence Detection System (Applied Biosystems, Foster City, CA, USA) were utilized for analysis, and relative expression levels of the vehicle control mice were calculated. The thermal cycling conditions were 10 min at 94 °C, followed by 39 cycles as follows: 15 s at 94 °C, 20 s at 57 °C, and 30 s at 72 °C. The sequences of the PCR oligonucleotide primers were as follows: PPARα, 5′- ATGCCAGTACTGCCGTTTTC-3′ and 5′-GGCCTTGACCTTGTTCATGT-3′; PPARγ, 5′- AGTGGAGACCGCCCAGG-3′ and 5′- GCAGCAGGTTGTCTTGGATGT-3′; Leptin, 5′- CCAAAACCCTCATCAAGACC-3′ and 5′- GTCCAACTGTTGAAGAATGTCCC-3′; UCP2, 5′- CCGCATTGGCCTCTACGACTCT-3′ and 5′- CCCCGAAGGCAGAAGTGAAGTG-3′; Adiponectin, 5′- CCCAAGGGAACTTGTGCAGGTTGGATG-3′ and 5′- GTTGGTATCATGGTAGAGAAGAAAGCC-3′; C/EBPα, 5′- TGGACAAGAACAGCAACGAGTAC-3′ and 5′- CGGTCATTGTCACTGGTCAACT-3′; C/EBPβ, 5′- AAGCTGAGCGACGAGTACAAGA-3′ and 5′- GTCAGCTCCAGCACCTTGTG-3′; SREBP1c, 5′- AGCCTGGCCATCTGTGAGAA-3′ and 5′- CAGACTGGTACGGGCCACAA-3′; FAS, 5′- GCTGCGGAAACTTCAGGAAAT-3′ and 5′- AGAGACGTGTCACTCCTGGACTT-3′; ACC1, 5′- GCCATTGGTATTGGGGCTTAC-3′ and 5′- CCCGACCAAGGACTTTGTTG-3′; AMPKα1, 5′- AAGCCGACCCAATGACATCA-3′ and 5′- CTTCCTTCGTACACGCAAAT-3′; AMPKα2, 5′- GATGATGAGGTGGTGGA-3′ and 5′-GCCGAGGACAAAGTGC-3′; GAPDH, 5′- CATCTTCCAGGAGCGAGACC-3′ and 5′- TCCACCACCCTGTTGCTGTA-3′. The data were normalized to GAPDH mRNA expression level by the comparative threshold cycle method [37].

### 2.11. Histopathology

Following the organ weights being measured, 10% neutral buffered formalin was used to fix the left lateral lobe of the liver, splenic lobe of the pancreas, left periovarian fat pads, and abdominal wall deposited fat pads stuck to the muscularis quadratus lumborum. After paraffin embedding had been carried out embedding by automated tissue processor (Shandon Citadel 2000, Thermo Scientific, Waltham, MA, USA) and embedding center (Shandon Histocentre 3, Thermo Scientific, Waltham, MA, USA), a microtome (RM2255, Leica Biosystems, Nussloch, Germany) was used to make serial sections. Once the sections were ready, the hematoxylin and eosin (HE) staining were performed for light microscopy analysis (Eclipse 80i; Nikon, Tokyo, Japan). The histological observations of each organ were conducted using a light microscope equipped with a camera system (ProgRes^TM^ C5, Jenoptik Optical Systems GmbH, Jena, Germany) and an automated image analyze program (*i*Solution FL ver 9.1, IMT *i*-solution Inc., Vancouver, British Columbia, Canada). The operator was blinded to the group allocation during this analysis. Otherwise, dehydrated liver portions, with 30% sucrose solution, were divided using a cryostat and stained with Oil Red-O. To observe additional details regarding hepatic changes, mean hepatocyte diameters (under H&E staining) were determined in the restricted view fields. The regions of steatohepatitis regions (i.e., proportion of fat placed in regions of the hepatic parenchyma) were determined as the proportions of lipid placed regions between the limited histological sight field of the liver using Oil Red-O staining. Average diameters of hepatocytes were determined in the limited sight field on a monitor on paraffin-embedded sections stained with H&E by an automatic image analysis procedure as μm. In addition, the mean diameters of white adipocytes in each fat pad were calculated. The thicknesses of the deposited periovarian and abdominal wall fat pads (mm) was determined, along with average areas occupied by zymogen granules. The numbers of pancreatic islets were also measured diameters of the pancreatic islets. Blinded evaluation was carried out by the histopathologist.

### 2.12. Immunohistochemistry

Using the avidin-biotin-peroxidase (ABC) detection method and guinea pig polyclonal insulin (Abcam, Cambridge, UK) or rabbit polyclonal glucagon (Abcam, Cambridge, UK) antiserum, serially sectioned pancreatic tissues were immunostained. Briefly, the incubation in methanol and 0.3% H_2_O_2_ for 30 min was performed to block the activity of endogenous peroxidase; Normal horse serum blocking solution (Vector Lab., Burlingame, CA, USA) in a humidity chamber for 1 h was used for non-specific immunoglobulin binding. This was followed by incubation with ABC reagents (Vectastain Elite ABC Kit, Vector Lab., Burlingame, CA, USA) and biotinylated universal secondary antibody (Vector Lab., Burlingame, CA, USA) in a humidity chamber for 1 h at room temperature. In the end, the sections were interacted with a peroxidase substrate kit (Vector Lab., Burlingame, CA, USA) at room temperature for 3 min. Between steps, all samples were rinsed three times using 0.01 M phosphate-buffered saline. Cells that exhibited more than 20% immunoreactivity (compared with other naïve cells) were defined as positive; the average numbers of glucagon- and insulin-immunoreactive cells scattered in 1 mm^2^ of pancreatic parenchyma were calculated by an automated imaging analysis program, and insulin/glucagon cell ratios were also calculated; Ratin of insulin/glucagon cells = (mean of insulin-immunoreactive cells/mean of glucagon-immunoreactive cells). When performing the analysis, the histopathologist was blinds to the group allocation.

### 2.13. Statistical Analyses

Variation of 8 mice were described as mean ± standard deviation (SD). Multiple comparison analysis for groups were performed. The Levene test was used for homogeneity of variance. If insignificance of homogeneity of variance was indicated by the Levene test, the one way analysis of variance (ANOVA) test was used as hypothesis testing, and then least-significant differences (LSD) post-hoc test was performed. If the Leven test indicated significance of homogeneity, Kruskal–Wallis H test was performed. When the Kruskal–Wallis H test indicated a significant variation between groups, the Mann–Whitney U (MW) test was used to determine the significant group difference. Statistical analyses were performed by SPSS (Ver. 14.0, IBM-SPSS Inc., Chicago, IL, USA).

## 3. Results

### 3.1. Effects of WL Against Obesity

#### 3.1.1. Effects of WL on Changes of Body Weight and Changes in Food Consumption

**Figure 1:**
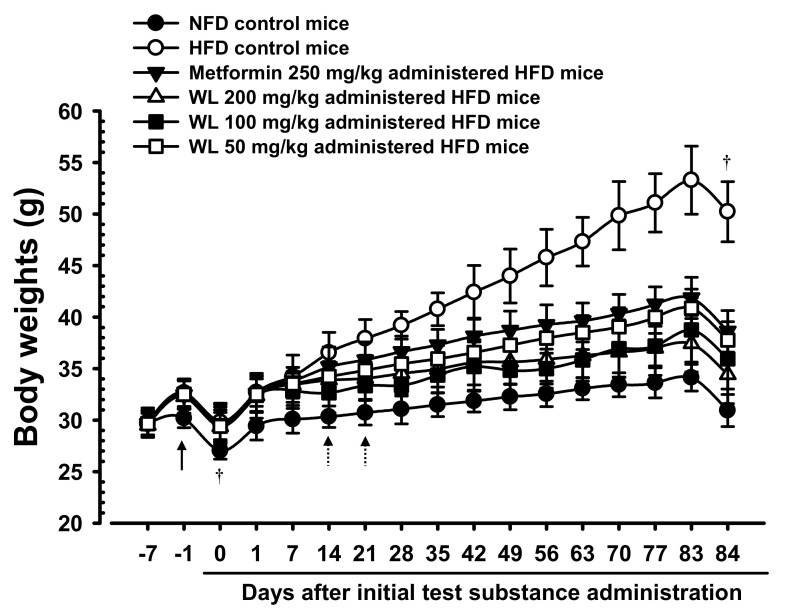
Body weight changes in NFD or HFD supplied mice. HFD control group showed significant (*p* < 0.01) increases of body weights as compared with intact group from 6 days after HFD supply (Arrow), however, significant (*p* < 0.01 or *p* < 0.05) decreases of the body weights were identified in all three different dosages of WL 200, 100, and 50 mg/kg groups from 14 days after first administration as compared with HFD control group, dose-dependently, and from 21 days after first administration in metformin group compared with HFD control group, respectively (Dot arrows). Values are shown as mean ± SD of eight mice. NFD = Normal pellet diet; HFD = 45% Kcal high-fat diet; WL = Wasabi Leaf/Folium, *Wasabia japonica* (Miq.) Matsum extracts. All animals were overnight fasted before first test material administrations and sacrifice (†). Day −7 means 7 days before initial test material administration; Day 0 means the day of initial test material administration; Day 84 means 1 day after last 84th test material administration.

The body weights significantly (*p* < 0.01) increased in HFD control group, compared with the weights in intact mice, beginning 6 days after the HFD had been first provided. Accordingly, the body weight changes during the HFD adaptation period and the test substances administration periods also significantly (*p* < 0.01) increased, compared with the body weight of intact control mice supplied the normal fat diet. However, significant (*p* < 0.01 or *p* < 0.05) dose-dependent reductions were detected in all three 200, 100, and 50 mg/kg WL dosage groups of mice beginning 14 days after first administration, compared with HFD control group, and from 21 days after first administration in 250 mg/kg metformin group, compared with HFD control group. Consequently, the body weight changes during administration period also decreased in a significant (*p* < 0.01) and dose-dependent manner in mice that received any WL treatment, as well as in metformin group, compared with changes in HFD control group. In particular, 50 mg/kg WL inhibited HFD-induced increases in body weight in a manner comparable to that of metformin (Table 1; Figure 1). The body weight changes during administration periods in HFD control group were increased 421.73% as compared to those of intact control group, but they were decreased −56.09, −75.20, −68.22, and −59.03% in metformin 250 mg/kg, WL 200, 100, and 50 mg/kg groups as compared to those of HFD control group.

Significant (*p* < 0.01) reduction of mean daily food consumption was detected in HFD control group, compared with the intact control group, however, insignificant variations in daily food consumption were observed in all test material-treated group, including those that received metformin, compared with HFD control mice (Table 1). The mean daily food consumptions during administration periods in HFD control group were decreased −16.99% as compared to intact control group, but they were changed by 0.32, −0.14, −0.04, and 0.71% in metformin 250 mg/kg, WL 200, 100, and 50 mg/kg groups as compared to those of HFD control group.

#### 3.1.2. Effects of WL on Body Fat Density

**Figure 2:**
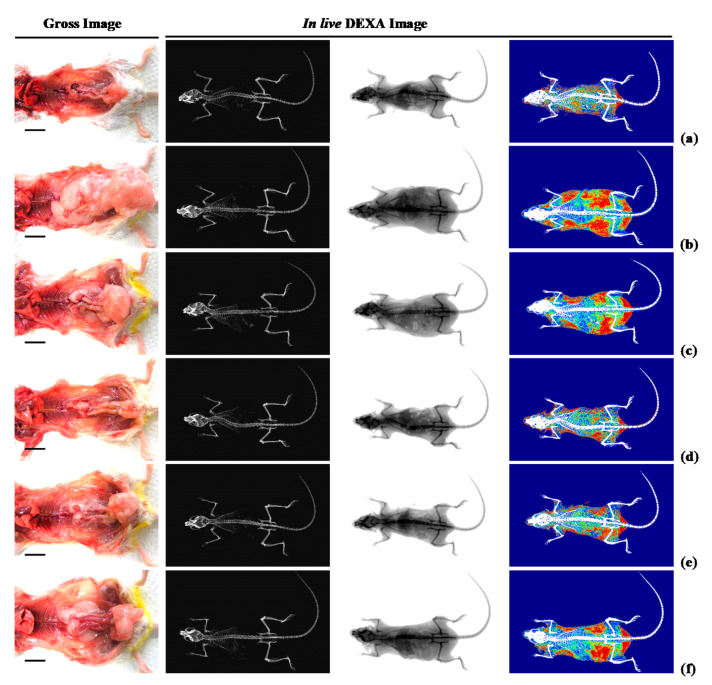
Representative gross body mass and abdominal fat pads with whole body DEXA images taken from NFD or HFD supplied mice. (**a**) Vehicle (distilled water) 10 mL/kg orally administered mice with NFD supply (Intact control); (**b**) Vehicle 10 mL/kg orally administered mice with HFD supply (HFD control); (**c**) 250 mg/kg of metformin oral administered mice with HFD supply (Metformin); (**d**) WL 200 mg/kg orally administered mice with HFD supply (WL200); (**e**) WL 100 mg/kg orally administered mice with HFD supply (WL100); (**f**) WL 50 mg/kg orally administered mice with HFD supply (WL50). Left column = bone; middle column = tissue; right column = composition (red = lipid; blue = protein). NFD = Normal pellet diet; HFD = 45% Kcal high-fat diet; WL = Wasabi Leaf/Folium, *Wasabia japonica* (Miq.) Matsum extracts; DEXA = Dual-energy X-ray absorptiometry. Scale bar = 12.8 mm.

**Figure 3:**
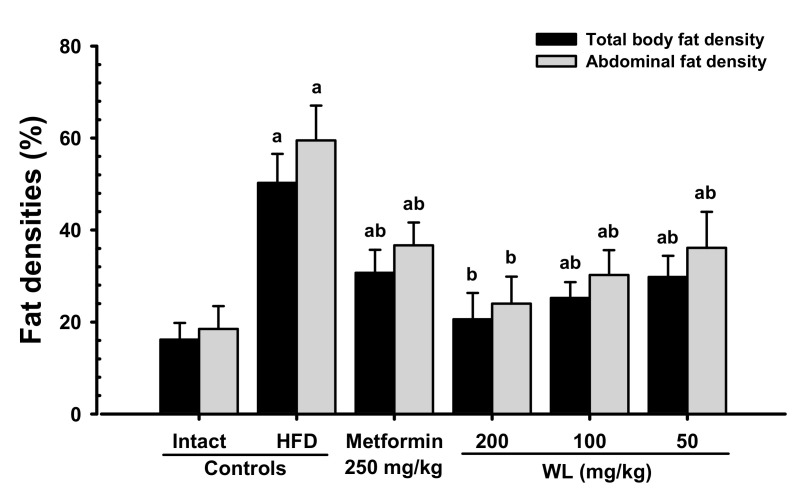
Total body and abdominal fat densities in NFD or HFD supplied mice. Values are shown as mean ± SD of eight mice. NFD = Normal pellet diet; HFD = 45% Kcal high-fat diet; WL = Wasabi Leaf/Folium, *Wasabia japonica* (Miq.) Matsum extracts. ^a^ *p* < 0.01—vs. intact control; ^b^ *p* < 0.01—vs. HFD control.

When compared with intact control group, HFD control group showed remarkable (*p* < 0.01) increases in both total body and abdominal fat densities. In contrast, significant (*p* < 0.01) dose-dependent reductions in both total body and abdominal fat width were observed at three WL dosages, compared with those parameters in HFD control and metformin group. In particular, 50 mg/kg WL and metformin inhibited the HFD-induced expansion in total body and abdominal fat densities (Figure 2 and Figure 3). The mean total body fat densities of HFD control group were increased 210.56% as compared to those of intact control group, but they were decreased −38.91, −59.03, −49.78 and −40.70% in metformin 250 mg/kg, WL 200, 100, and 50 mg/kg groups as compared to those of HFD control group. The mean abdominal fat densities of HFD control were increased 221.25% as compared to those of intact control group, but they were decreased −38.33, −59.65, −49.18 and −39.24% in metformin 250 mg/kg, WL 200, 100, and 50 mg/kg groups as compared to HFD control group.

#### 3.1.3. Effects of WL on Periovarian and Abdominal Wall Deposited Fat Pad Weights

When compared with intact control group, HFD control group showed significant (*p* < 0.01) increases in periovarian and abdominal wall deposited fat pad weights. However, the increases in periovarian fat pad weights were reduced in dose-dependent manner (*p* < 0.01) in response to 250 mg/kg metformin treatment and each of the three 200, 100, and 50 mg/kg WL dosages, compared with the results in HFD control mice group. In particular, 50 mg/kg WL and metformin inhibited the HFD-induced increases in periovarian and abdominal wall deposited fat pad weights (Table 2; Figure 2). The absolute periovarian fat pad weights in HFD control group were increased 916.67% as compared to intact control group, but they were decreased −61.56, −80.90, −70.07, and −61.85% in metformin 250 mg/kg, WL 200, 100, and 50 mg/kg controls as compared to those of HFD control. The relative periovarian fat pad weights in HFD control group were increased 528.77% as compared to those of intact control group, but they were decreased −49.83, −72.52, −57.89, and −49.24% in metformin 250 mg/kg, WL 200, 100, and 50 mg/kg groups as compared to those of HFD control group. The absolute abdominal wall deposited fat pad weights in HFD control group were increased 1091.25% as compared to those of intact control group, but they were decreased −61.28, −77.33, −70.93, and −62.71% in metformin 250 mg/kg, WL 200, 100, and 50 mg/kg groups as compared to HFD control group. The relative abdominal wall deposited fat pad weights in HFD control group were increased 635.54% as compared to intact control group, but they were decreased −49.72, −67.46, −59.71, and −50.42% in metformin 250 mg/kg, WL 200, 100, and 50 mg/kg groups as compared to those of HFD control group.

#### 3.1.4. Effects of WL on Adipocyte Histopathology

**Figure 4:**
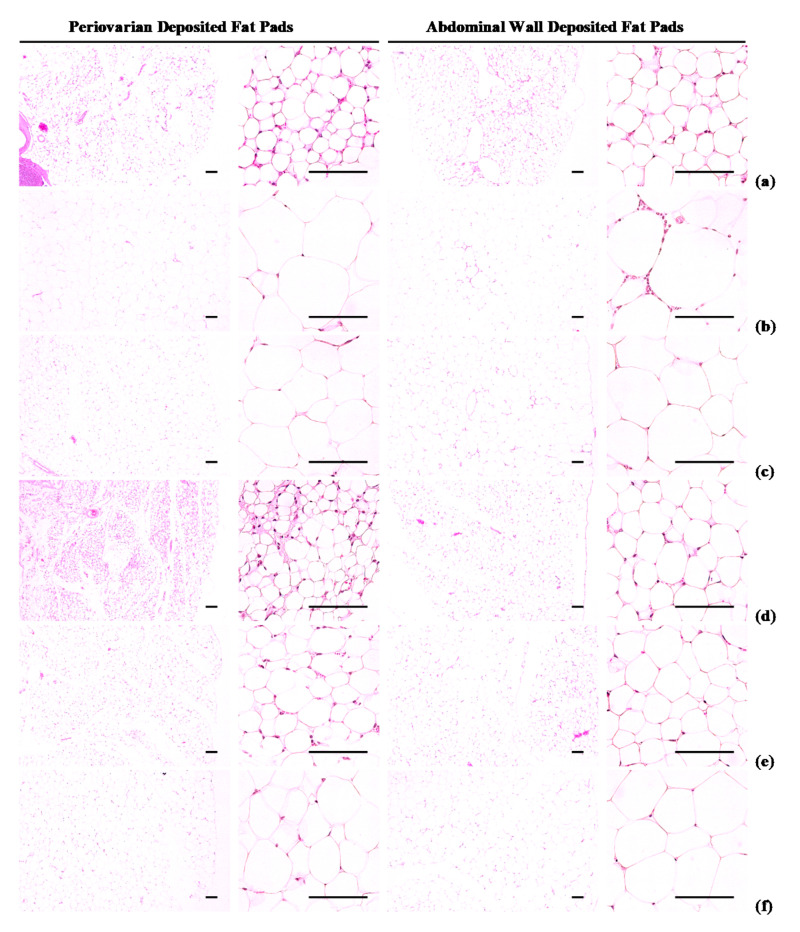
Representative histological images of the adipocytes, taken from NFD or HFD supplied mice periovarian and abdominal wall deposited fat pads. (**a**) Vehicle (distilled water) 10 mL/kg orally administered mice with NFD supply (Intact control); (**b**) Vehicle 10 mL/kg orally administered mice with HFD supply (HFD control); (**c**) 250 mg/kg of metformin oral administered mice with HFD supply (Metformin); (**d**) WL 200 mg/kg orally administered mice with HFD supply (WL200); (**e**) WL 100 mg/kg orally administered mice with HFD supply (WL100); (**f**) WL 50 mg/kg orally administered mice with HFD supply (WL50). NFD = Normal pellet diet; HFD = 45% Kcal high-fat diet; WL = Wasabi Leaf/Folium, *Wasabia japonica* (Miq.) Matsum extracts. All Hematoxylin & Eosin stain. Scale bars = 80 µm.

Periovarian and abdominal thicknesses of deposited fat pads and white adipocyte diameters were higher (*p* < 0.01) in HFD control group than in intact control group. However, hypertrophy of the adipocytes and fat deposits was inhibited in a significant (*p* < 0.01) and dose-dependent manner by each of the three WL dosages and by metformin, compared with the results in HFD control mice. In particular, 50 mg/kg WL and metformin inhibited the HFD-induced changes in histopathological fat pad depositions and adipocyte hypertrophy (Table 3; Figure 4). The deposited periovarian fat pad thicknesses in HFD control group were increased 286.18% as compared to those of intact control group, but they were decreased −49.84, −66.48, −61.14, and −51.62% in metformin 250 mg/kg, WL 200, 100, and 50 mg/kg groups as compared to HFD control group. The mean periovarian white adipocyte diameters in HFD control group were increased 235.21% as compared to intact control group, but they were changed by −39.34, −63.31, −54.43, and −40.47% in metformin 250 mg/kg, WL 200, 100, and 50 mg/kg groups as compared to those of HFD control group. The abdominal wall deposited fat pad thicknesses in HFD control were increased 454.29% as compared to those of intact control group, but they were decreased −52.59, −73.83, −67.58, and −53.85% in metformin 250 mg/kg, WL 200, 100, and 50 mg/kg groups as compared to those of HFD control group. The mean abdominal wall deposited fat pad white adipocyte diameters in HFD control group were increased 181.53% as compared to intact control, but they were decreased −36.84, −56.22, −49.75, and −38.48% in metformin 250 mg/kg, WL 200, 100, and 50 mg/kg groups as compared to HFD control group.

#### 3.1.5. Effects of WL on Exocrine Pancreas Zymogen Granule Content

**Figure 5:**
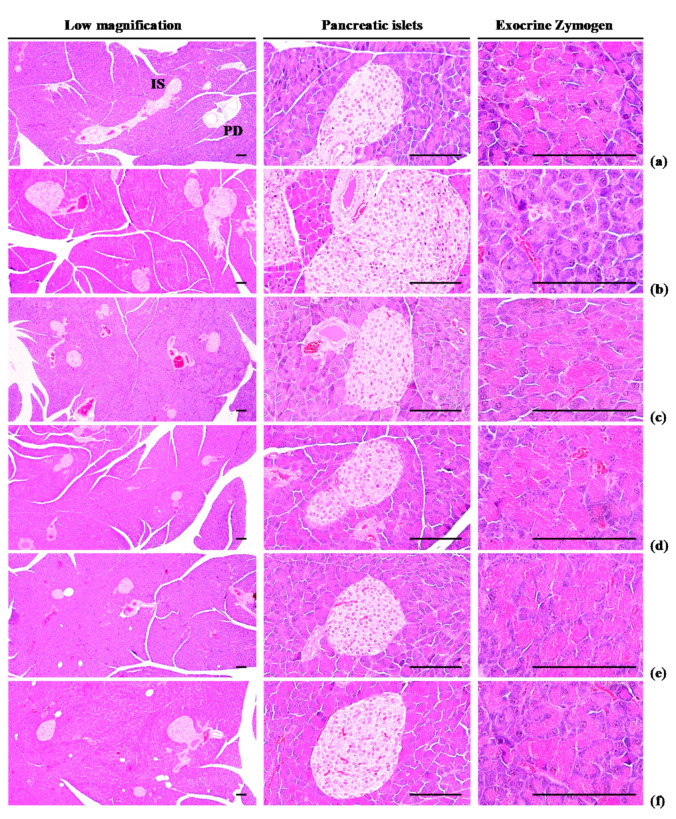
Representative general histological images of the pancreas, taken from NFD or HFD supplied mice. (**a**) Vehicle (distilled water) 10 mL/kg orally administered mice with NFD supply (Intact control); (**b**) Vehicle 10 mL/kg orally administered mice with HFD supply (HFD control); (**c**) 250 mg/kg of metformin oral administered mice with HFD supply (Metformin); (**d**) WL 200 mg/kg orally administered mice with HFD supply (WL200); (**e**) WL 100 mg/kg orally administered mice with HFD supply (WL100); (**f**) WL 50 mg/kg orally administered mice with HFD supply (WL50). NFD = Normal pellet diet; HFD = 45% Kcal high-fat diet; WL = Wasabi Leaf/Folium, *Wasabia japonica* (Miq.) Matsum extracts; IS = Pancreatic islet; PD = Pancreatic secretory duct. All Hematoxylin & Eosin stain. Scale bars = 80 µm.

Exocrine pancreas zymogen granule content was less (*p* < 0.01) in HFD control group than in intact control group, due to the discharge of zymogen granules. However, exocrine pancreas zymogen granule content significantly (*p* < 0.01) increased in three WL dosage groups and the metformin group, compared with the results in HFD control group. In particular, 50 mg/kg WL and metformin inhibited the HFD-induced exocrine pancreas zymogen granule depletions at histopathological levels (Table 4; Figure 5). The proportion regions of exocrine pancreas filled by zymogen granule in HFD control group were decreased −78.08% as compared to intact control group, but they were increased 210.39, 309.07, 259.93, and 217.69% in metformin 250 mg/kg, WL 200, 100, and 50 mg/kg groups as compared to those of HFD control group.

### 3.2. Anti-Diabetic Hypoglycemic Effects

#### 3.2.1. Effects on Blood Glucose and Serum Insulin Levels and Blood HbA1c Content

**Figure 6:**
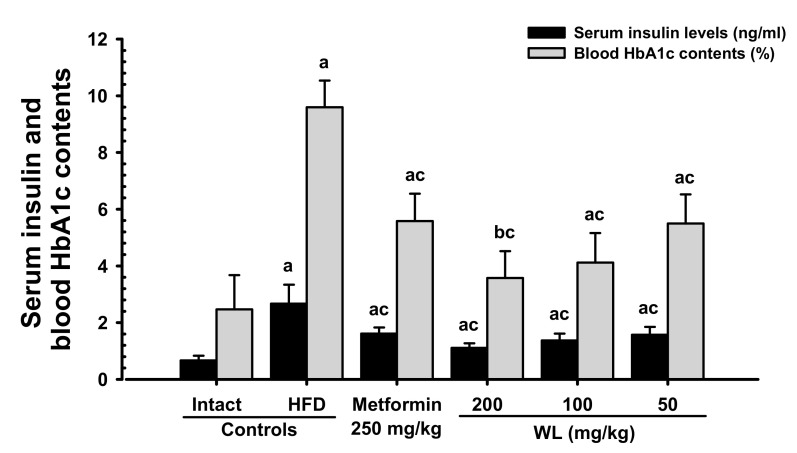
Serum insulin and blood HbA1c contents in NFD or HFD supplied mice. Values are shown as mean ± SD of eight mice. NFD = Normal pellet diet; HFD = 45% Kcal high-fat diet; WL = Wasabi Leaf/Folium, *Wasabia japonica* (Miq.) Matsum extracts; HbA1c = Glycated hemoglobin, hemoglobin A1c. ^a^ *p* < 0.01 and ^b^ *p* < 0.05—vs. intact control; ^c^ *p* < 0.01—vs. HFD control.

Blood HbA1c content, serum glucose, and insulin levels were significantly (*p* < 0.01) increased in HFD control group compared to the intact control group. However, serum glucose and insulin levels, as well as blood HbA1c content, dose-dependently (*p* < 0.01) decreased in response to metformin treatment and each of the three WL dosages, compared with the results in HFD control group. In particular, 50 mg/kg WL and metformin inhibited the HFD-induced elevations in blood glucose, serum insulin level, and blood HbA1c content (Table 5; Figure 6). The blood glucose levels in HFD control group were increased 171.94% as compared to those of intact control group, but they were decreased −36.68, −52.72, −46.19, and −37.29% in metformin 250 mg/kg, WL 200, 100, and 50 mg/kg groups as compared to those of HFD control group. The serum insulin levels in HFD control group were increased 299.07% as compared to intact control group, but they were decreased −39.53, −58.36, −48.52, and −41.08% in metformin 250 mg/kg, WL 200, 100, and 50 mg/kg groups as compared to HFD control group. The blood HbA1c contents in HFD control group were increased 288.91% as compared to intact control group, but they were decreased −41.83, −62.75, −57.08, and −42.73% in metformin 250 mg/kg, WL 200, 100, and 50 mg/kg groups as compared to those of HFD control group.

#### 3.2.2. Effects of WL on Pancreatic Weights

Significant (*p* < 0.01) reductions in relative weights of the pancreas were detected in HFD control group, compared with intact control group. However, significant (*p* < 0.01) dose-dependent increases in relative pancreatic weights were detected in mice taking each of the three WL dosages, and in the metformin group, compared with the results in HFD control group. No changes in absolute weights of the pancreas were demonstrated in any of the HFD-supplied mice, including HFD control group, compared with intact control group. There were no changes in absolute weights of pancreas in HFD control group and intact control group (Table 6). The absolute weights of the pancreas in HFD control group were decreased −8.27% as compared to those of intact control group, but they were increased 2.49, 9.63, 7.58, and 1.44% in metformin 250 mg/kg, WL 200, 100, and 50 mg/kg groups as compared to those of HFD control group. The relative weights of pancreas in HFD control group were decreased −43.47% as compared to intact control group, but they were increased 33.12, 61.34, 51.02, and 35.44% in metformin 250 mg/kg, WL 200, 100, and 50 mg/kg groups as compared to HFD control group.

#### 3.2.3. Effects of WL on Pancreatic Islet Hyperplasia and Expansion

Pancreatic islet mean diameters and numbers were significantly (*p* < 0.01) higher observed in HFD control group than intact control group because of marked pancreatic islet hyperplasia or component endocrine cells. Hyperplasia and expansion of islets decreased in a significant and dose-dependent manner (*p* < 0.01) in response to metformin treatment and each of the three WL dosages, compared with the results in HFD control group. In particular, 50 mg/kg WL and metformin inhibited the HFD-induced hyperplasia and expansion of pancreatic islets (Table 7; Figure 5). The mean pancreatic islet numbers in HFD control group were increased 344.74% as compared to those of intact control group, but they were changed by −48.52, −71.01, −62.72, and −49.11% in metformin 250 mg/kg, WL 200, 100, and 50 mg/kg groups as compared to HFD control group. The proportions of islet filled regions in HFD control group were increased 117.85% as compared to intact control group, but they were decreased −43.60, −53.28, −48.47, and −43.90% in metformin 250 mg/kg, WL 200, 100, and 50 mg/kg groups as compared to HFD control group.

#### 3.2.4. Effects of WL on Pancreatic Islet Glucagon- and Insulin-Immunoreactive Cells

**Figure 7:**
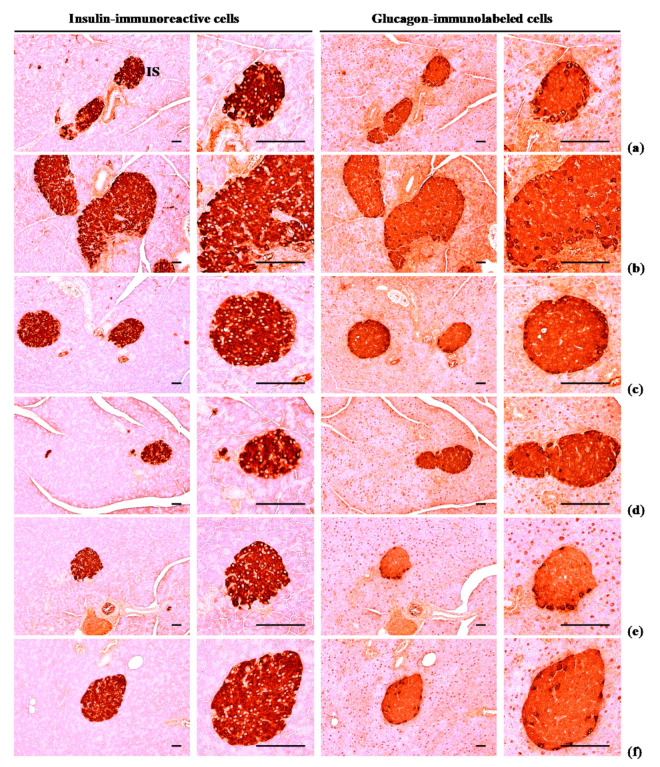
Representative histological images of the insulin- and glucagon-immunoreactive cells in the pancreas, taken from NFD or HFD supplied mice. (**a**) Vehicle (distilled water) 10 mL/kg orally administered mice with NFD supply (Intact control); (**b**) Vehicle 10 mL/kg orally administered mice with HFD supply (HFD control); (**c**) 250 mg/kg of metformin oral administered mice with HFD supply (Metformin); (**d**) WL 200 mg/kg orally administered mice with HFD supply (WL200); (**e**) WL 100 mg/kg orally administered mice with HFD supply (WL100); (**f**) WL 50 mg/kg orally administered mice with HFD supply (WL50). NFD = Normal pellet diet; HFD = 45% Kcal high-fat diet; WL = Wasabi Leaf/Folium, *Wasabia japonica* (Miq.) Matsum extracts; IS = Pancreatic islet. All immunostained by avidin-biotin-peroxidase complex. Scale bars = 80 µm.

The numbers of glucagon and insulin-immunoreactive cells and ration of insulin/glucagon cells were higher (*p* < 0.01) in HFD control group than in intact control group. Though, these abnormal increases in glucagon and insulin-immunostained cells and their ratio were normalized in a significant and dose-dependent (*p* < 0.01) manner by each of the three WL dosages and by metformin, compared with the results in HFD control group. In particular, 50 mg/kg WL and metformin inhibited the HFD-induced hyperplasia of insulin and glucagon-immunoreactive cells, and increased insulin/glucagon cells (Table 8; Figure 7). The mean numbers of insulin-immunoreactive cells in HFD control group were increased 1117.60% as compared to intact control group, but they were decreased −50.87, −86.63, −75.35, and −53.46% in metformin 250 mg/kg, WL 200, 100, and 50 mg/kg groups as compared to HFD control group. The mean numbers of glucagon-immunolabeled cells in HFD control group were increased 532.74% as compared to those of intact control group, but they were decreased −28.83, −76.50, −61.19, and −30.56% in metformin 250 mg/kg, WL 200, 100, and 50 mg/kg groups as compared to HFD control group. The ratio of insulin/glucagon cells in HFD control group was increased 89.87% as compared to intact control group, but it was decreased −29.52, −42.26, −36.58, and −32.61% in metformin 250 mg/kg, WL 200, 100, and 50 mg/kg groups as compared to those of HFD control group.

### 3.3. Effects on Hyperlipidemia

#### 3.3.1. Effects on Serum Total Cholesterol, TG, LDL, and HDL-C Levels

Significant (*p* < 0.01) increases in serum TG, total cholesterol, and LDL levels, as well as reductions in HDL-C levels, were observed in HFD control group, compared with intact control group. However, serum TG, total cholesterol, and LDL levels significantly (*p* < 0.01) decreased, while serum HDL-C levels increased in a significant and dose-dependent (*p* < 0.01) manner in all three WL dosage groups and the metformin group, compared with the results in HFD control group. In particular, 50 mg/kg WL and metformin inhibited the HFD-induced increases in serum TG, total cholesterol, LDL levels, and decreases in HDL-C levels (Table 9). The serum total cholesterol levels in HFD control group were increased 222.33% as compared to those of intact control group, but they were decreased −39.63, −56.28, −45.35, and −39.41% in metformin 250 mg/kg, WL 200, 100, and 50 mg/kg groups as compared to those of HFD control group. The serum TG levels in HFD control group were increased 232.50% as compared to intact control group, but they were decreased −42.72, −58.59, −50.48, and −42.67% in metformin 250 mg/kg, WL 200, 100, and 50 mg/kg groups as compared to those of HFD control group. The serum LDL levels in HFD control group were increased 351.52% as compared to those of intact control group, but they were decreased −36.58, −61.91, −50.67, and −38.09% in metformin 250 mg/kg, WL 200, 100, and 50 mg/kg groups as compared to HFD control group. The serum HDL-C levels in HFD control group were decreased −77.04% as compared to intact control, but they were increased 151.20, 271.08, 222.89, and 150.00% in metformin 250 mg/kg, WL 200, 100, and 50 mg/kg group as compared to HFD control group.

#### 3.3.2. Effects on Fecal Total Cholesterol and TG Contents

**Figure 8:**
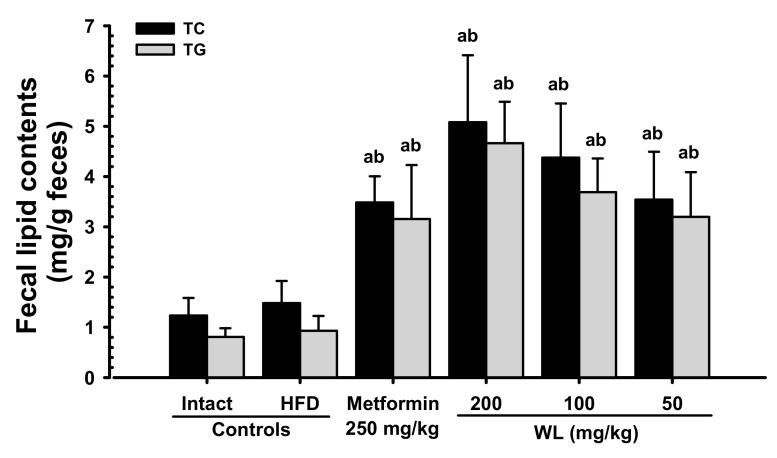
Fecal total cholesterol and TG content in NFD or HFD supplied mice. Values are shown as mean ± SD of eight mice. NFD = Normal pellet diet; HFD = 45% Kcal high-fat diet; WL = Wasabi Leaf/Folium, *Wasabia japonica* (Miq.) Matsum extracts; TG = Triglyceride. ^a^ *p* < 0.01—vs. intact control; ^b^ *p* < 0.01—vs. HFD control.

Though slight non-significant increases in fecal total cholesterol and TG contents were observed in HFD control group, compared with intact control group, the fecal total cholesterol and TG contents in three WL dosage groups and the metformin group increased in a significant and dose-dependent (*p* < 0.01) manner, compared with the results in HFD control group. In particular, 50 mg/kg WL and metformin showed favorable effects on fecal total cholesterol and TG contents, their excretions (Figure 8). The fecal total cholesterol contents in HFD control group were increased 19.92% as compared to those of intact control group, but they were changed by 135.08, 242.75, 195.28, and 138.87% in metformin 250 mg/kg, WL 200, 100, and 50 mg/kg groups as compared to HFD control group. The fecal TG contents in HFD control group were increased 15.15% as compared to intact control group, but they were changed by 238.79, 400.87, 296.11, and 243.36% in metformin 250 mg/kg, WL 200, 100, and 50 mg/kg groups as compared to those of HFD control group.

### 3.4. Effects on Hepatopathy

#### 3.4.1. Effects on Liver Weight

Significant (*p* < 0.01) increases in absolute weights of liver were observed in HFD control group, compared with intact control group; however, these increments were normalized in a significant and dose-dependent (*p* < 0.01) manner by administration with each of the three WL dosages and by metformin, compared with the results in HFD control group. In particular, 50 mg/kg WL and metformin inhibited the HFD-induced decreases in liver absolute weight. No changes in relative weights of the liver were demonstrated in any of the experimental HFD-fed mice, including HFD control group, compared with intact control group. Insignificant changes in relative weights of the liver were detected in any of the test material-treated mice, including those that received metformin, compared with the results in HFD control group (Table 10). The absolute liver weights in HFD control group were increased 69.34% as compared to those of intact control group, but they were decreased -24.53, −36.43, −33.98, and −24.87% in metformin 250 mg/kg, WL 200, 100, and 50 mg/kg groups as compared to those of HFD control group.

#### 3.4.2. Effects on Serum ALT, AST, GGT, ALP, and LDH levels

Significant (*p* < 0.01) increments in serum ALT, AST, GGT, ALP, and LDH levels were identified in HFD control group, compared with intact control group. However, serum ALT, AST, GGT, ALP, and LDH levels decreased in a significant and dose-dependent (*p* < 0.01) manner in response to metformin treatment and each of the three WL dosages, compared with the results in HFD control group. In particular, 50 mg/kg WL and metformin inhibited the HFD-induced increases in serum ALT, AST, GGT, ALP, and LDH levels (Table 11). The serum AST levels in HFD control group were increased 201.30% as compared to intact control group, but they were decreased −36.37, −56.25, −47.41, and −36.69% in metformin 250 mg/kg, WL 200, 100, and 50 mg/kg groups as compared to HFD control group. The serum ALT levels in HFD control group were increased 299.39% as compared to those of intact control group, but they were deceased −35.42, −55.73, −45.50, and −33.66% in metformin 250 mg/kg, WL 200, 100, and 50 mg/kg groups as compared to HFD control group. The serum ALP levels in HFD control group were increased 188.69% as compared to intact control group, but they were decreased −38.26, −59.04, −50.70, and −40.63% in metformin 250 mg/kg, WL 200, 100, and 50 mg/kg groups as compared to those of HFD control group. The serum LDH levels in HFD control group were increased 598.57% as compared to those of intact control group, but they were decreased −60.06, −78.07, −68.06, and −60.02% in metformin 250 mg/kg, WL 200, 100, and 50 mg/kg groups as compared to those of HFD control group. The serum GGT levels in HFD control group were increased 251.43% as compared to intact control group, but they were decreased −52.03, −63.41, −58.54, and −51.22% in metformin 250 mg/kg, WL 200, 100, and 50 mg/kg groups as compared to HFD control group.

#### 3.4.3. Effects on Steatosis and Hepatocyte Hypertrophy

**Figure 9:**
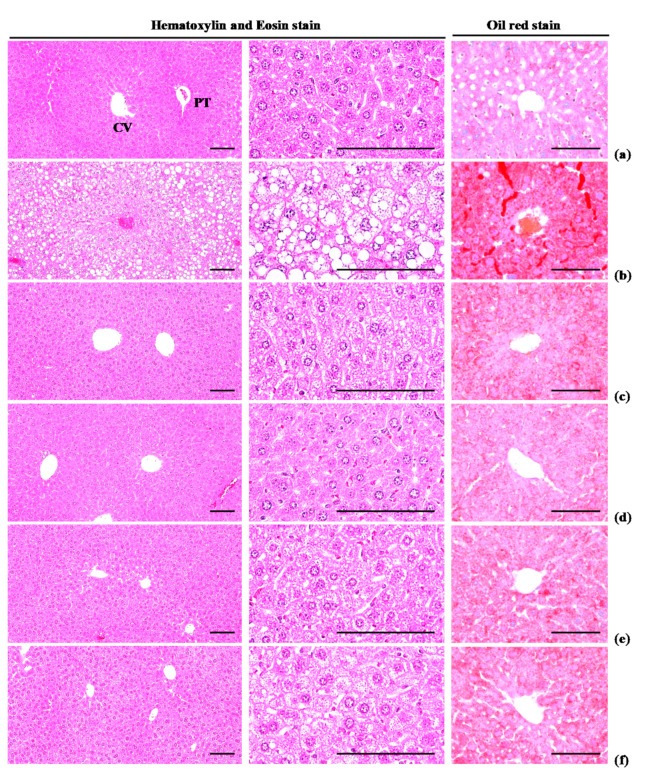
Representative histological images of the liver, taken from NFD or HFD supplied mice. (**a**) Vehicle (distilled water) 10 mL/kg orally administered mice with NFD supply (Intact control); (**b**) Vehicle 10 mL/kg orally administered mice with HFD supply (HFD control); (**c**) 250 mg/kg of metformin oral administered mice with HFD supply (Metformin); (**d**) WL 200 mg/kg orally administered mice with HFD supply (WL200); (**e**) WL 100 mg/kg orally administered mice with HFD supply (WL100); (**f**) WL 50 mg/kg orally administered mice with HFD supply (WL50). NFD = Normal pellet diet; HFD = 45% Kcal high-fat diet; WL = Wasabi Leaf/Folium, *Wasabia japonica* (Miq.) Matsum extracts; CV = Central vein; PT = Portal triad. Scale bars = 80 µm.

Significant (*p* < 0.01) increases in steatosis (percentages of fatty changed areas in liver parenchyma) and mean hepatocyte diameter (hypertrophy) were observed in HFD control group, compared with intact control group, due to serious hypertrophy of hepatocytes relative to intracellular lipid deposits. Oil Red-O stained histopathological steatosis and hepatocyte hypertrophy were normalized in a significant and dose-dependent (*p* < 0.01) manner by treatment with each of the three WL dosages and by metformin, compared with the results in HFD control mice. In particular, 50 mg/kg WL and metformin inhibited the HFD-induced steatosis at histopathological levels (Table 12; Figure 9). The steatosis regions in HFD control group were increased 911.57% as compared to those of intact control group, but they were decreased −52.51, −73.75, −64.14, and −53.22% in metformin 250 mg/kg, WL 200, 100, and 50 mg/kg groups as compared to those of HFD control group. The mean hepatocyte diameters in HFD control group were increased 101.66% as compared to intact control group, but they were decreased −31.39, −41.41, −39.76, and −33.47% in metformin 250 mg/kg, WL 200, 100, and 50 mg/kg groups as compared to HFD control group.

### 3.5. Effects on Liver Antioxidant Defense System and Lipid Peroxidation

Hepatic malondialdehyde content and liver lipid peroxidation were higher (*p* < 0.01) in HFD control group than in intact control group; however, these elevations were normalized in a significant and dose-dependent (*p* < 0.01) manner by administration with each of the three WL dosages and by metformin, compared with the results in HFD control group. In particular, 50 mg/kg WL and metformin inhibited the HFD-induced hepatic lipid peroxidation (Table 13). The hepatic lipid peroxidation in HFD control group was increased 670.48% as compared to intact control group, but it was decreased −48.37, −71.70, −62.78, and −51.38% in metformin 250 mg/kg, WL 200, 100, and 50 mg/kg groups as compared to HFD control group.

Significant (*p* < 0.01) reductions in hepatic CAT, SOD activities, and GSH contents were detected in HFD control group, compared with intact control group. Hepatic CAT, SOD activities, and GSH contents increased in a significant and dose-dependent (*p* < 0.01) manner in response to metformin treatment and each of the three WL dosages, compared with the results in HFD control group. In particular, 50 mg/kg WL and metformin inhibited the HFD-induced decreases in hepatic GSH, CAT, and SOD activities (Table 14). The hepatic GSH contents in HFD control group were decreased −86.45% as compared to those of intact control group, but they were increased 254.60, 543.83, 444.57, and 285.32% in metformin 250 mg/kg, WL 200, 100, and 50 mg/kg groups as compared to those of HFD control group. The hepatic CAT activities in HFD control group were decreased −83.96% as compared to intact control group, but they were increased 219.65, 389.59, 315.13, and 225.14% in metformin 250 mg/kg, WL 200, 100, and 50 mg/kg groups as compared to those of HFD control group. The hepatic SOD activities in HFD control group were decreased −90.62% as compared to those of intact control group, but they were increased 320.42, 677.83, 493.09, and 331.08% in metformin 250 mg/kg, WL 200, 100, and 50 mg/kg groups as compared to HFD control group.

### 3.6. Effects on Hepatic Glucose-Regulating Enzyme Activities

Significant (*p* < 0.01) reductions in hepatic GK activity and increases in hepatic G6pase and PEPCK activities were observed in HFD control group, compared with intact control group; though, these changes were normalized in a significant and dose-dependent (*p* < 0.01) manner after administration with each of the three WL dosages and by metformin, compared with the results in HFD control group. In particular, 50 mg/kg WL and metformin inhibited the HFD-induced decreases in hepatic GK activity, and increased in hepatic G6pase and PEPCK activities (Table 15). The hepatic GK activities in HFD control group were decreased −71.70% as compared to intact control group, but they were increased 96.29, 141.16, 112.60, and 100.43% in metformin 250 mg/kg, WL 200, 100, and 50 mg/kg groups as compared to HFD control group. The hepatic G6pase activities in HFD control group were increased 177.85% as compared to those of intact control group, but they were decreased −45.04, −53.01, −50.72, and −45.50% in metformin 250 mg/kg, WL 200, 100, and 50 mg/kg groups as compared to HFD control group. The hepatic PEPCK activities in HFD control group were increased 261.93% as compared to intact control group, but they were decreased −51.98, −66.33, −60.08, and −52.91% in metformin 250 mg/kg, WL 200, 100, and 50 mg/kg groups as compared to those of HFD control group.

### 3.7. Effects on Lipid Metabolism-Related Gene Expression

Significant (*p* < 0.01) increases in hepatic ACC1 and adipose tissue leptin, C/EBPα, C/EBPβ, fatty acid synthase, SREBP1c, and PPARγ mRNA expression levels, as well as reductions in hepatic AMPKα1 and AMPKα2 expression levels were observed in HFD control group. In addition, adipose tissue UCP2, adiponectin, and PPARα mRNA expression levels were lower in HFD control group than in intact control group. Notably, these changes were normalized in a significant and dose-dependent (*p* < 0.01) manner after administration with each of the three WL dosages and by metformin, compared with the results in HFD control group. In particular, 50 mg/kg WL and metformin inhibited the HFD-induced abnormal hepatic ACC1, AMPKα1, and AMPKα2 mRNA expression, and abnormal adipose tissue leptin, C/EBPα, C/EBPβ, FAS, SREBP1, PPARγ, UCP2, adiponectin, and PPARα mRNA expression changes (Table 16).

The hepatic ACC1 mRNA expressions in HFD control group were increased 435.08% as compared to those of intact control group, but they were decreased −50.54, −73.97, −63.91, and −52.29% in metformin 250 mg/kg, WL 200, 100, and 50 mg/kg groups as compared to those of HFD control group. The hepatic AMPKα1 mRNA expressions in HFD control group were decreased −75.28% as compared to those of intact control group, but they were increased 130.46, 212.69, 169.04, and 137.56% in metformin 250 mg/kg, WL 200, 100, and 50 mg/kg groups as compared to those of HFD control group. The hepatic AMPKα2 mRNA expressions in HFD control group were decreased −78.21% as compared to those of intact control group, but they were increased 137.14, 245.71, 202.86, and 148.57% in metformin 250 mg/kg, WL 200, 100, and 50 mg/kg groups as compared to those of HFD control group.

The adipose tissue leptin mRNA expressions in HFD control group were increased 704.13% as compared to those of intact control group, but they were decreased −60.35, −82.65, −73.82, and −61.14% in metformin 250 mg/kg, WL 200, 100, and 50 mg/kg groups as compared to those of HFD control group. The adipose tissue UCP2 mRNA expressions in HFD control group were decreased −78.31% as compared to those of intact control group, but they were increased 147.43, 265.14, 226.29, and 149.14% in metformin 250 mg/kg, WL 200, 100, and 50 mg/kg groups as compared to those of HFD control group. The adipose tissue adiponectin mRNA expressions in HFD control group were decreased −84.72% as compared to those of intact control group, but they were increased 203.25, 327.64, 281.30, and 204.07% in metformin 250 mg/kg, WL 200, 100, and 50 mg/kg groups as compared to those of HFD control group. The adipose tissue C/EBPα mRNA expressions in HFD control group were increased 180.12% as compared to those of intact control group, but they were decreased −43.06, −55.84, −48.66, and −42.71% in metformin 250 mg/kg, WL 200, 100, and 50 mg/kg groups as compared to those of HFD control group. The adipose tissue C/EBPβ mRNA expressions in HFD control group were increased 299.25% as compared to those of intact control group, but they were decreased −45.34, −66.34, −50.75, and −47.96% in metformin 250 mg/kg, WL 200, 100, and 50 mg/kg groups as compared to those of HFD control group. The adipose tissue SREBP1c mRNA expressions in HFD control group were increased 174.31% as compared to those of intact control group, but they were decreased −40.45, −57.90, −50.14, and −41.83% in metformin 250 mg/kg, WL 200, 100, and 50 mg/kg groups as compared to those of HFD control group. The adipose tissue PPARα mRNA expressions in HFD control group were decreased −83.17% as compared to those of intact control group, but they were increased 167.41, 331.85, 265.93, and 182.96% in metformin 250 mg/kg, WL 200, 100, and 50 mg/kg groups as compared to those of HFD control group. The adipose tissue PPARγ mRNA expressions in HFD control group were increased 755.40% as compared to those of intact control group, but they were decreased −46.60, −72.61, −55.15, and −48.23% in metformin 250 mg/kg, WL 200, 100, and 50 mg/kg groups as compared to those of HFD control group. The adipose tissue FAS mRNA expressions in HFD control group were increased 1388.40% as compared to those of intact control group, but they were decreased −65.91, −80.97, −75.57, and −66.70% in metformin 250 mg/kg, WL 200, 100, and 50 mg/kg groups as compared to those of HFD control group.

## 4. Discussion

**Figure 10:**
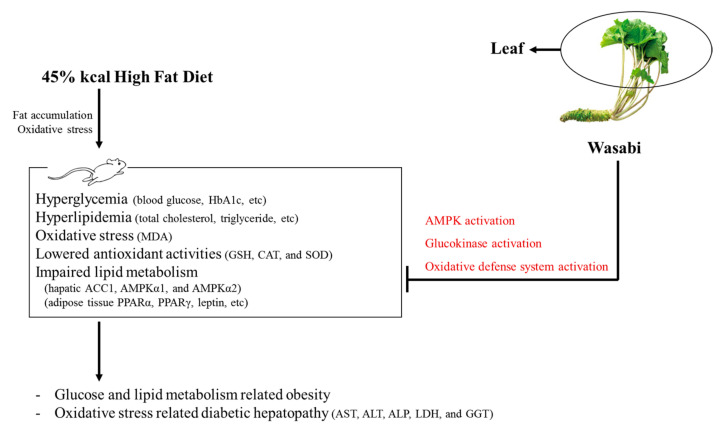
The comprehensive results of Wasabi Leaf against diabetic obesity in the present study. HbA1c = Glycated hemoglobin; MDA = Malondialdehyde; GSH = Glutathione; CAT = Catalase; SOD = Superoxide dismutase; ACC1 = Acetyl-CoA carboxylase 1; AMPK = 5’ adenosine monophosphate-activated protein kinase; PPAR = Peroxisome proliferator-activated receptor.

All diabetes manifestations and related complications, including NAFLD and obesity, were dose-dependently inhibited in following 84 days of continuous oral treatment with three WL dosages. In particular, 50 mg/kg WL showed consistent remarkable reduction against HFD-induced diabetes and related complications (e.g., obesity, NAFLD, nephropathy, and hyperlipidemia) that were similar to those of 250 mg/kg metformin. These results indicate that 50 mg/kg WL mediates remarkable effects on diabetes and related complications by upregulation of AMPK-related hepatic glucose enzyme and lipid metabolism-related gene expression compared to metformin 250 mg/kg. The 50 mg/kg WL also elevated the modulation of pancreatic lipid enzyme and activation of antioxidant defense system compared to metformin 250 mg/kg (Figure 10).

An increase in the deposit of adipose tissue is a general aspect of obesity [13,14,16,31]. Adipose tissue acts as an energy storage organ, as well as an endocrine and secretory organ [38]. Adipose tissue secretes adipokines; alterations in the action, expression, and secretion of adipokines in obese individuals may contribute to the development of various diseases, including insulin resistance [16,39]. In the present study, 84 days continuous oral treatment of all three dosages of WL 200, 100, and 50 mg/kg, dose-dependently and significantly inhibited the deposit of adipose tissues and adipocyte hypertrophy, and also by metformin 250 mg/kg. Especially, WL 50 mg/kg constantly showed favorable inhibitory activities against HFD-induced accumulation of adipose tissues and adipocyte hypertrophy as comparable to metformin group, at least in a condition of the present DEXA and histopathological analysis. These results are considered as obvious evidences that WL has favorable anti-obese activities in HFD mice, dose-dependently, and constantly as comparable to metformin 250 mg/kg, at dose level of 50 mg/kg (Table 2 and Table 3, Figure 2, Figure 3 and Figure 4). It is known that obese individuals develop a reduced number of zymogen granules, acinar cell atrophy, and pancreatic steatosis [14,16,31,40]. The increase of pancreatic zymogen granules in exocrine acinar cells implies the production of digestive enzymes, particularly for digestion of protein and lipid [41]. The diminishments of pancreatic zymogen granules were also detected in HFD control group as compared with intact control group at histopathological observation, and they caused lipid absorption related obesity. Though, the treatment of all three dosage of WL effectively inhibited the diminishments of zymogen depositions in exocrine pancreas, dose-dependently, and also by metformin 250 mg/kg. In particular, WL 50 mg/kg constantly showed affirmative inhibitory activities against HFD-induced exocrine pancreas zymogen granule, in the current study. These results are considered as evidences that WL has favorable anti-obese activities, might be through pancreatic enzyme production or release inhibitions in HFD supplied mice, dose-dependently, and constantly as comparable to metformin treatment, at dose of 50 mg/kg, at least in a condition of the present analysis (Table 4, Figure 5).

Glycated hemoglobin (HbA1c) is used to determine the average concentration of plasma glucose over elongated periods of time; importantly, HbA1c is formed when erythrocytes are exposed to high glucose concentrations [42,43]. Hyperglycemia is the key cause and symptom of diabetes; this parameter should be regulated to treat diabetes [13,16]. HFD-fed mice have been wildly employed to study for type II diabetes; they have been shown to develop noticeable hyperglycemia [13,14,29,31]. As insulin resistance progresses to type II diabetes, enhanced levels in blood insulin and Hb1Ac have been detected after chronic intake of an HFD [44,45]. In addition, increment of insulin secretion is partly associated with pancreatic islet hyperplasia during the development of insulin resistance [46]. The total numbers of pancreatic islets and insulin-producing cells increased after mice had been fed the HFD. The islets increased in number and area to secrete more insulin, presumably to preserve glucose homeostasis [47], along with obvious hyperplasia or hypertrophy of endocrine pancreatic cells [46,47]. In addition, in the current experiment, obvious increases of blood glucose, HbA1c, and insulin contents were defected in HFD control group as compared with intact control group, and with increments of pancreatic islet expansions, numbers, glucagon- and insulin-immunoreactive cells, and their ratios, indicating insulin resistance type II diabetes at histopathological observations. Though, 84 days oral administration of three dosages of WL significantly inhibited these abnormal histopathological changes of endocrine pancreas and increments of blood glucose, HbA1c, and insulin contents, dose-dependently, and also by metformin treatment. In particular, WL 50 mg/kg constantly showed affirmative inhibitory activities against HFD-induced insulin resistance type II diabetes as similar to metformin 250 mg/kg in the current study. These results are considered as reliable evidences that WL has affirmative hypoglycemic effects may be through the pancreatic endocrine change inhibition effects in HFD supplied mice, dose-dependently, and as comparable to metformin 250 mg/kg at dose of 50 mg/kg, at least in a condition of this analysis (Figure 6).

Decreases in serum LDL, TG, and total cholesterol, as well as increases in HDL levels, are generally observed during progression of chronic diabetes in HFD-fed mice [13,15,16]. In the present study, oral administration of three dosages of WL actively decreased the serum LDL, TG and total cholesterol levels, and elevated the serum HDL-C levels. In particular, WL 50 mg/kg constantly presented affirmative inhibitory activities against HFD-induced hyperlipidemia as comparable to metformin 250 mg/kg in the current serum biochemistrical analysis. These results are considered as reliable evidences that WL has affirmative hypolipidemic effects may be through the inhibitory activities on the pancreatic enzyme production or releases in HFD supplied mice, dose-dependently, and as similar to metformin 250 mg/kg at oral dose of 50 mg/kg, at least in a condition of our analysis. In addition, these favorable hypolipidemic effects of WL, at doses of 200, 100, and 50 mg/kg, observed in these HFD mice were also regarded as results from the decrements of lipid absorptions and secretion of lipids into feces, through the pancreatic digestive enzyme regulating effects as previously described. Once again, marked and significant increments of total cholesterol and TG contents in feces were demonstrated after treatment of three dosages of WL, dose-dependently, and also by metformin treatment as compared to those of HFD control group. In particular, WL 50 mg/kg showed affirmative effects on fecal total cholesterol and TG contents, their excretions, as similar to metformin 250 mg/kg, at least in a condition of the current observation (Table 9, Figure 8). As diabetes progressed, fibrosis induced the increments of liver weight or lipid deposition in the cytoplasm induced abnormal glycosylation related to hepatosteatosis, and hypertrophic changes in hepatocytes were observed along with increases in serum ALT, AST, GGT, ALP, and LDH levels [13,16]. These phenomena are regarded as diabetic hepatopathy; they also manifest as NAFLD in HFD-fed mice [13,14,31,48]. Improvements in these abnormalities are direct evidence to support improvement of diabetic hepatopathy. AST is primarily located in the liver and striated muscle. Serum AST activity increases with higher levels of skeletal muscle necrosis and hepatocellular necrosis. Elevation of serum AST activity, with no increase in ALT, indicates muscle necrosis; however, activity of AST is higher than ALT in the context of liver damage, suggesting more broad cellular destruction because AST spills from necrotic cells, rather than cells with an unstable membrane [49]. ALT is mainly located in the cytoplasm of hepatocytes. If liver cells are damaged or destroyed, ALT enters the blood, and then circulates for a few days. Increases in serum AST and ALT levels are responsive indicators of liver damage, as are increases in serum ALP, LDH, and GGT; however, changes in ALP, LDH, and GGT do not indicate the reason or reversibility of the damage [49]. In this experiment, 84 days continuous oral administration of all three different dosages of WL effectively decreased the diabetic hepatopathies, dose-dependently, and also by metformin 250 mg/kg. In particular, WL 50 mg/kg constantly showed affirmative inhibitory activities against HFD-induced diabetic hepatopathies as comparable to metformin 250 mg/kg, in the current analysis. These results are considered as reliable evidences that WL has affirmative hepatoprotective effects in HFD supplied mice, dose-dependently, and as similar to metformin 250 mg/kg at oral dose of 50 mg/kg, at least in a condition of the present observation (Table 11).

Accumulating evidence indicates the roles of free radicals in the pathogenesis of diabetes and impaired antioxidant defense [50]. Oxidative stress plays a key role in the etiology of diabetes mellitus. The hyperglycemia-induced free radicals generation is highly associated with glucose auto-oxidation. Notably, glucose auto-oxidation has been known to be linked to non-enzymatic glycosylation; glycosylated proteins are a source of ROS [16,51]. Oxidative stress in subjects with diabetes occurs with a diminishing antioxidant defense [52], which elevates the damaging effects of free radicals. The increased production of ROS-related oxidative stress plays a key role in the pathogenesis of diabetic problems [53]. Numerous lipid peroxidation products damage to the nearby tissues [54]; HFD-fed mice displayed elevated lipid peroxidation in several organs, that they performed as a potent redox cycler to generate harmful ROS that damaged organs [12,55]. GSH prevents tissue damage by maintenance of ROS at low levels [56]; in addition, SOD is an antioxidant enzyme that plays to enzymatic defense, while CAT is an enzyme that catalyzes the conversion of H_2_O_2_ to H_2_O [57]. Thus, the enhancement of lipid peroxidation and reductions of endogenous antioxidants, GSH content, and SOD and CAT activities that occur in damaged liver tissue are secondary considerations in terms of improving diabetes and related problems [58,59]. In the current study, HFD control mice displayed an elevated lipid peroxidation, reduced GSH content, and decreased activities of SOD and CAT in the liver [44,60]. In the present study, treatment of three dosages of WL effectively inhibited the decline of hepatic antioxidant defense systems, and also by metformin treatment. In particular, WL 50 mg/kg constantly showed affirmative inhibitory activities against HFD-induced decline of hepatic antioxidant defense systems and related lipid peroxidation as similar to metformin 250 mg/kg, in the current detection. These results are considered as obvious evidences that WL has affirmative antioxidant effects in HFD supplied mice, dose-dependently, and as comparable to metformin 250 mg/kg at a dose of 50 mg/kg, at least in a condition of this observation (Table 13 and Table 14)

The hepatic enzyme GK is included in the control of glucose homeostasis; its elevated increased expression can enhance blood glucose application for energy production or glycogen storage in the liver, causing a decreased blood glucose level [61,62]. In contrast, G6pase and PEPCK are related with gluconeogenesis and hepatic glucose output; their elevated activities indicate an increment in the blood glucose level [63,64]. Noticeable reductions in liver GK activities with elevates in G6pase and PEPCK activities were present in both HFD-fed mice [44] and HFD control group in the present study. Constantly, treatment of three dosages of WL effectively inhibited the HFD-induced hepatic glucose-regulating enzyme activity changes, and also by metformin treatment. Especially, WL 50 mg/kg constantly showed affirmative inhibitory activities against HFD-induced hepatic glucose-regulating enzyme activity changes as similar to metformin 250 mg/kg, in the current inspection. These results are considered as clear evidences that WL has affirmative hepatic glucose-regulating enzyme activity modulatory effects in HFD supplied mice, dose-dependently, and as similar to metformin 250 mg/kg at dose of 50 mg/kg, enough to be developed as an ingredient of medicinal food and natural drugs for obese type II diabetes through hepatic glucose-regulating enzyme regulations, at least in a condition of our observation (Table 15).

We investigated lipid metabolism-related gene mRNA expression in hepatic and adipose tissues to elucidate the mechanisms by which the test material exerted anti-diabetic effects on related complications, including NAFLD and obesity. Regulation of glucose and lipid homeostasis through the activation of fatty acid oxidation and the suppression of lipogenesis and glucose production is influenced by AMPK activation [65,66]. Considering the role of AMPK signaling pathway—in lipid and glucose metabolism, it is essential to analyze their mRNA and protein expression levels in adipose tissue and the liver. In addition, PPARα performs a pivotal role in the stimulation of beta-oxidation, while PPARγ is involved in activation of genes for *de novo* lipogenesis [36,67]. UCP2 is a representative thermogenesis-related protein [68]; the effects of fat cell-secreted adiponectin on insulin sensitizing and fatty-acid-oxidizing actions is rely on AMPK in adiponectin in adipose tissue and liver [69,70]. In addition, upregulation of AMPK and the AMPK signaling pathway consistently showed beneficial modulatory activities on the endogenous antioxidant defense system [14,71] and glucose-regulatory enzyme activities [66,72]. Therefore, we investigated whether the test material affected mRNA expression of AMPK and AMPK signaling pathway related-proteins in the liver and adipose tissues. Our results showed that AMPKα and AMPKα2 mRNA expression levels were reduced in the liver of HFD-fed mice, indicating that changes in AMPK expression are involved in the pathogenesis of lipid accumulation and NAFLD [35]. In addition, significant increases of hepatic ACC1 mRNA expressions, adipose tissue C/EBPα, C/EBPβ, FAS, SREBP1c, leptin, and PPARγ mRNA expressions were also observed in HFD control group, with significant decrements of adipose tissue UCP2, adiponectin, and PPARα mRNA expressions. However, these HFD-induced abnormal lipid metabolism related gene mRNA expressions were significantly normalized by oral administration of three dosages of WL, and also by metformin treatment. In particular, WL 50 mg/kg constantly showed affirmative inhibitory activities against HFD-induced abnormal AMPK and lipid metabolism related gene mRNA expressions as similar to metformin 250 mg/kg, in the present *realtime* RT-PCR analysis. These results are considered as definitive evidences that WL has affirmative AMPK up-regulation mediated suppressing lipogenesis and promoting fatty acid oxidation related lipid metabolism improvement activities in HFD supplied mice, dose-dependently, and as similar to metformin 250 mg/kg at oral dose of 50 mg/kg, enough to be developed as an ingredient of medicinal food and natural drugs for obese type II diabetes through AMPK and lipid metabolism related gene mRNA expression modulations, at least in a condition of the present analysis (Table 16).

## 5. Conclusions

The present study demonstrated that 50 mg/kg WL had favorable effects on diabetes and related complications (e.g., obesity, NAFLD, and hyperlipidemia) in HFD-fed mice, in a manner comparable to treatment with 250 mg/kg metformin; these effects were mediated by upregulation of AMPK-related hepatic glucose enzyme activities and lipid metabolism-related gene expression, the antioxidant defense system, and pancreatic lipid digestion enzyme modulatory activities.

## Figures and Tables

**Table 1:** Changes in body weight and mean daily food consumption in NFD or HFD supplied mice.

Times Groups	Body Weights (g) at Days after First Test Material Treatment				Body Weight Gains During		Mean Daily Food Consumption (g)
	7 Days Before [A]	1 Day Before [B]	0 Day * [C]	84 Days * [D]	Adapt Period [B–A]	Administration Period [D–C]	
Controls							
Intact	29.83 ± 1.05	30.19 ± 0.93	27.03 ± 0.81	30.94 ± 1.56	0.56 ± 0.48	3.91 ± 1.29	6.07 ± 0.25
HFD	29.79 ± 1.41	32.66 ± 1.34 ^a^	29.81 ± 1.70 ^a^	50.23 ± 2.91 ^a^	2.88 ± 0.77 ^a^	20.41 ± 2.10 ^a^	5.04 ± 0.15 ^a^
Reference							
Metformin	29.66 ± 1.24	32.48 ± 1.42 ^a^	29.66 ± 1.98 ^a^	38.63 ± 2.02 ^ac^	2.81 ± 0.70 ^a^	8.96 ± 1.98 ^ac^	5.06 ± 0.22 ^a^
Test materials—WL							
200 mg/kg	29.50 ± 1.16	32.29 ± 1.61 ^a^	29.36 ± 1.91 ^a^	34.43 ± 2.81 ^ac^	2.79 ± 1.17 ^a^	5.06 ± 2.14 ^c^	5.03 ± 0.20 ^a^
100 mg/kg	29.66 ± 1.33	32.53 ± 1.39 ^a^	29.46 ± 1.54 ^a^	35.95 ± 2.92 ^ac^	2.86 ± 0.86 ^a^	6.49 ± 2.15 ^bc^	5.04 ± 0.35 ^a^
50 mg/kg	29.65 ± 1.15	32.50 ± 1.32 ^a^	29.40 ± 1.34 ^a^	37.76 ± 1.79 ^ac^	2.85 ± 0.68 ^a^	8.36 ± 1.62 ^ac^	5.08 ± 0.28 ^a^

Values are shown as mean ± SD of eight mice. NFD = Normal pellet diet; HFD = 45% Kcal high-fat diet; WL = Wasabi Leaf/Folium, *Wasabia japonica* (Miq.) Matsum extracts. A = Seven days before the test material administration; B = One day before the test material administration; C = Start of the test material administration; D = End of 84 days continuous oral administration. * All animals were overnight fasted. ^a^ *p* < 0.01 and ^b^ *p* < 0.05—vs. intact control; ^c^ *p* < 0.01—vs. HFD control.

**Table 2:** Variations on absolute and relative fat pads weights in NFD or HFD supplied mice.

Groups Items	Control		Reference	Test Materials—WL		
	Intact	HFD	Metformin	200 mg/kg	100 mg/kg	50 mg/kg
Absolute organ weights (g)						
Periovarian fat pads	0.056 ± 0.021	0.564 ± 0.127 ^a^	0.217 ± 0.038 ^ac^	0.108 ± 0.043 ^bc^	0.169 ± 0.033 ^ac^	0.215 ± 0.026 ^ac^
Abdominal wall fat pads	0.050 ± 0.023	0.596 ± 0.111 ^a^	0.231 ± 0.059 ^ac^	0.135 ± 0.064 ^bc^	0.173 ± 0.061 ^ac^	0.222 ± 0.036 ^ac^
Relative organ weights (% of body weights)						
Periovarian fat pads	0.179 ± 0.066	1.123 ± 0.242 ^a^	0.563 ± 0.110 ^ac^	0.309 ± 0.116 ^bc^	0.473 ± 0.103 ^ac^	0.570 ± 0.065 ^ac^
Abdominal wall fat pads	0.161 ± 0.074	1.187 ± 0.218 ^a^	0.597 ± 0.146 ^ac^	0.386 ± 0.183 ^ac^	0.478 ± 0.160 ^ac^	0.588 ± 0.094 ^ac^

Values are shown as mean ± SD of eight mice. NFD = Normal pellet diet; HFD = 45% Kcal high-fat diet; WL = Wasabi Leaf/Folium, *Wasabia japonica* (Miq.) Matsum extracts. ^a^ *p* < 0.01 and ^b^ *p* < 0.05—vs. intact control; ^c^ *p* < 0.01—vs. HFD control.

**Table 3:** The histopathologic and histomorphometric changes of the periovarian and abdominal wall fat pads in NFD or HFD supplied mice.

Items Groups	Periovarian Fat Pads		Abdominal Wall Fat Pads	
	Thickness (mm)	Adipocyte Diameters (μm)	Thickness (mm)	Adipocyte Diameters (μm)
Controls				
Intact	1.73 ± 0.67	32.25 ± 1.94	1.31 ± 0.50	39.95 ± 3.81
HFD	6.67 ± 1.24 ^a^	108.12 ± 14.91 ^a^	7.28 ± 1.15 ^a^	112.47 ± 12.26 ^a^
Reference				
Metformin	3.35 ± 1.09 ^ac^	65.58 ± 12.01 ^ac^	3.45 ± 0.83 ^ac^	71.04 ± 10.58 ^ac^
Test materials—WL				
200 mg/kg	2.24 ± 0.48 ^c^	39.67 ± 6.80 ^c^	1.90 ± 0.25 ^bc^	49.24 ± 10.85 ^c^
100 mg/kg	2.59 ± 0.46 ^bc^	49.27 ± 10.54 ^ac^	2.36 ± 0.56 ^ac^	56.52 ± 13.82 ^ac^
50 mg/kg	3.23 ± 0.75 ^ac^	64.37 ± 16.38 ^ac^	3.36 ± 1.27 ^ac^	69.19 ± 15.50 ^ac^

Values are shown as mean ± SD of eight mice. NFD = Normal pellet diet; HFD = 45% Kcal high-fat diet; WL = Wasabi Leaf/Folium, *Wasabia japonica* (Miq.) Matsum extracts. ^a^ *p* < 0.01 and ^b^ *p* < 0.05—vs. intact control; ^c^ *p* < 0.01—vs. HFD control.

**Table 4:** Changes in pancreas zymogen granule content in NFD or HFD supplied mice.

Groups Items	Control		Reference	Test Materials—WL		
	Intact	HFD	Metformin	200 mg/kg	100 mg/kg	50 mg/kg
Zymogen granules (%/mm^2^ of exocrine)	59.72 ± 11.66	13.09 ± 3.29 ^a^	40.62 ± 11.08 ^ac^	53.54 ± 10.91 ^c^	47.11 ± 10.54 ^bc^	41.58 ± 12.69 ^ac^

Values are shown as mean ± SD of eight mice. NFD = Normal pellet diet; HFD = 45% Kcal high-fat diet; WL = Wasabi Leaf/Folium, *Wasabia japonica* (Miq.) Matsum extracts. ^a^ *p* < 0.01 and ^b^ *p* < 0.05—vs. intact control; ^c^ *p* < 0.01—vs. HFD control.

**Table 5:** Changes in blood glucose levels in NFD or HFD supplied mice.

Groups Items	Control		Reference	Test Materials—WL		
	Intact	HFD	Metformin	200 mg/kg	100 mg/kg	50 mg/kg
Glucose (mg/dL)	97.13 ± 27.25	264.13 ± 34.78 ^a^	167.25 ± 21.33 ^ac^	124.88 ± 14.65 ^bc^	142.13 ± 13.73 ^ac^	165.63 ± 29.02 ^ac^

Values are shown as mean ± SD of eight mice. NFD = Normal pellet diet; HFD = 45% Kcal high-fat diet; WL = Wasabi Leaf/Folium, *Wasabia japonica* (Miq.) Matsum extracts. ^a^ *p* < 0.01 and ^b^ *p* < 0.05—vs. intact control; ^c^ *p* < 0.01—vs. HFD control.

**Table 6:** Changes in absolute and relative pancreas weight in NFD or HFD supplied mice.

Groups Items	Control		Reference	Test Materials—WL		
	Intact	HFD	Metformin	200 mg/kg	100 mg/kg	50 mg/kg
Absolute organ weights (g)	0.246 ± 0.028	0.226 ± 0.029	0.232 ± 0.028	0.248 ± 0.026	0.243 ± 0.024	0.229 ± 0.017
Relative organ weights (% of body weights)	0.796 ± 0.075	0.450 ± 0.054 ^a^	0.599 ± 0.059 ^ac^	0.726 ± 0.114 ^c^	0.679 ± 0.084 ^ac^	0.609 ± 0.068 ^ac^

Values are shown as mean ± SD of eight mice. NFD = Normal pellet diet; HFD = 45% Kcal high-fat diet; WL = Wasabi Leaf/Folium, *Wasabia japonica* (Miq.) Matsum extracts. ^a^ *p* < 0.01 —vs. intact control; ^c^ *p* < 0.01—vs. HFD control.

**Table 7:** Changes in pancreatic islet numbers and mean diameters in NFD or HFD supplied mice.

Groups Items	Control		Reference	Test Materials—WL		
	Intact	HFD	Metformin	200 mg/kg	100 mg/kg	50 mg/kg
Mean islet numbers (numbers/10 mm^2^)	4.75 ± 1.49	21.13 ± 2.23 ^a^	10.88 ± 1.96 ^ac^	6.13 ± 1.13 ^c^	7.88 ± 1.36 ^ac^	10.75 ± 3.15 ^ac^
Mean islet diameter (μm/islet)	122.11 ± 19.56	266.01 ± 32.40 ^a^	150.04 ± 14.67 ^bc^	124.29 ± 13.37 ^c^	137.08 ± 10.77 ^c^	149.24 ± 15.40 ^bc^

Values are shown as mean ± SD of eight mice. NFD = Normal pellet diet; HFD = 45% Kcal high-fat diet; WL = Wasabi Leaf/Folium, *Wasabia japonica* (Miq.) Matsum extracts. ^a^ *p* < 0.01 and ^b^ *p* < 0.05—vs. intact control; ^c^ *p* < 0.01—vs. HFD control.

**Table 8:** Changes in histopathology-histomorphometry of the pancreas in NFD or HFD supplied mice.

Items Groups	Insulin-IR Cells (Cells/mm^2^) [A]	Glucagon-IR Cells (Cells/mm^2^) [B]	Insulin/Glucagon Ratio [A/B]
Controls			
Intact	260.63 ± 25.67	84.75 ± 14.08	3.12 ± 0.34
HFD	3173.38 ± 547.90 ^a^	536.25 ± 88.39 ^a^	5.92 ± 0.42 ^a^
Reference			
Metformin	1559.13 ± 129.66 ^ac^	381.63 ± 72.27 ^ac^	4.17 ± 0.56 ^ac^
Test materials—WL			
200 mg/kg	424.13 ± 115.96 ^ac^	126.00 ± 43.94 ^ac^	3.42 ± 0.34 ^c^
100 mg/kg	782.25 ± 109.94 ^ac^	208.13 ± 20.57 ^ac^	3.75 ± 0.34 ^ac^
50 mg/kg	1476.75 ± 265.78 ^ac^	372.38 ± 63.49 ^ac^	3.99 ± 0.53 ^ac^

Values are shown as mean ± SD of eight mice. NFD = Normal pellet diet; HFD = 45% Kcal high-fat diet; WL = Wasabi Leaf/Folium, *Wasabia japonica* (Miq.) Matsum extracts; IR = Immunoreactive. A = Number of insulin immunoreactive cell; B = Number of glucagon immunoreactive cell. ^a^ *p* < 0.01 —vs. intact control; ^c^ *p* < 0.01—vs. HFD control.

**Table 9:** Changes in blood glucose levels and serum lipid contents in NFD or HFD supplied mice.

Items Groups	Total Cholesterol (mg/dL)	Triglyceride (mg/dL)	Low Density Lipoprotein (mg/dL)	High Density Lipoprotein-Cholesterol(mg/dL)
Controls				
Intact	90.13 ± 15.42	74.63 ± 20.26	16.50 ± 2.83	90.38 ± 16.15
HFD	290.50 ± 23.72 ^a^	248.13 ± 29.07 ^a^	74.50 ± 17.39 ^a^	20.75 ± 3.37 ^a^
Reference				
Metformin	175.38 ± 21.76 ^ac^	142.13 ± 16.21 ^ac^	47.25 ± 10.31 ^ac^	52.13 ± 13.82 ^ac^
Test materials—WL				
200 mg/kg	127.00 ± 22.88 ^ac^	102.75 ± 14.50 ^bc^	28.38 ± 10.49 ^bc^	77.00 ± 10.90 ^c^
100 mg/kg	158.75 ± 23.63 ^ac^	122.88 ± 22.79 ^ac^	36.75 ± 12.73 ^ac^	67.00 ± 19.26 ^ac^
50 mg/kg	176.00 ± 25.93 ^ac^	142.25 ± 25.38 ^ac^	46.13 ± 11.92 ^ac^	51.88 ± 14.48 ^ac^

Values are shown as mean ± SD of eight mice. NFD = Normal pellet diet; HFD = 45% Kcal high-fat diet; WL = Wasabi Leaf/Folium, *Wasabia japonica* (Miq.) Matsum extracts. ^a^ *p* < 0.01 and ^b^ *p* < 0.05—vs. intact control; ^c^ *p* < 0.01—vs. HFD control.

**Table 10:** Changes in absolute and relative liver weight in NFD or HFD supplied mice.

Groups Items	Control		Reference	Test Materials—WL		
	Intact	HFD	Metformin	200 mg/kg	100 mg/kg	50 mg/kg
Absolute organ weights (g)	1.194 ± 0.088	2.022 ± 0.213 ^a^	1.526 ± 0.105 ^ac^	1.286 ± 0.051 ^bc^	1.335 ± 0.050 ^ac^	1.519 ± 0.113 ^ac^
Relative organ weights (% of body weights)	3.863 ± 0.264	4.028 ± 0.388	3.962 ± 0.363	3.752 ± 0.272	3.735 ± 0.324	4.023 ± 0.239

Values are shown as mean ± SD of eight mice. NFD = Normal pellet diet; HFD = 45% Kcal high-fat diet; WL = Wasabi Leaf/Folium, *Wasabia japonica* (Miq.) Matsum extracts. ^a^ *p* < 0.01 and ^b^ *p* < 0.05—vs. intact control; ^c^ *p* < 0.01—vs. HFD control.

**Table 11:** Changes in serum AST, ALT, ALP, LDH, and GGT levels in NFD or HFD supplied mice.

Items Groups	AST (IU/L)	ALT (IU/L)	ALP (IU/L)	LDH (× 10 IU/L)	GGT (IU/L)
Controls					
Intact	77.00 ± 13.53	41.00 ± 13.01	77.38 ± 19.71	56.95 ± 15.83	4.38 ± 2.39
HFD	232.00 ± 37.45 ^a^	163.75 ± 20.36 ^a^	223.38 ± 43.58 ^a^	397.83 ± 107.86 ^a^	15.38 ± 3.20 ^a^
Reference					
Metformin	147.63 ± 21.73 ^ac^	105.75 ± 19.17 ^ac^	137.88 ± 19.65 ^ac^	158.91 ± 27.34 ^ac^	7.38 ± 1.77 ^ac^
Test materials—WL					
200 mg/kg	101.50 ± 19.77 ^bc^	72.50 ± 18.54 ^ac^	91.50 ± 14.92 ^c^	87.26 ± 24.09 ^bc^	5.63 ± 1.30 ^c^
100 mg/kg	122.00 ± 22.21 ^ac^	89.25 ± 18.98 ^ac^	110.13 ± 15.37 ^bc^	127.08 ± 18.59 ^ac^	6.38 ± 1.92 ^c^
50 mg/kg	146.88 ± 16.42 ^ac^	108.63 ± 18.38 ^ac^	132.63 ± 23.17 ^ac^	159.03 ± 27.15 ^ac^	7.50 ± 2.00 ^ac^

Values are shown as mean ± SD of eight mice. NFD = Normal pellet diet; HFD = 45% Kcal high-fat diet; WL = Wasabi Leaf/Folium, *Wasabia japonica* (Miq.) Matsum extracts; AST = Aspartate aminotransferase; ALT = Alanine aminotransferase; ALP = Alkaline phosphatase; LDH = Lactate dehydrogenase; GGT = Gamma glutamyl transferase. ^a^ *p* < 0.01 and ^b^ *p* < 0.05—vs. intact control; ^c^ *p* < 0.01—vs. HFD control.

**Table 12:** Changes in histopathology-histomorphometry of the liver in NFD or HFD supplied mice.

Items Groups	Liver Steatosis (%/mm^2^ of Hepatic Tissues)	Mean Hepatocyte Diameters (μm/cell)
Controls		
Intact	8.12 ± 2.15	17.32 ± 2.83
HFD	82.10 ± 11.04 ^a^	34.94 ± 1.82 ^a^
Reference		
Metformin	38.99 ± 12.58 ^ac^	23.97 ± 3.11 ^ac^
Test materials—WL		
200 mg/kg	21.55 ± 10.09 ^bc^	20.47 ± 1.35 ^ac^
100 mg/kg	29.44 ± 10.23 ^ac^	21.05 ± 1.34 ^ac^
50 mg/kg	38.41 ± 10.18 ^ac^	23.24 ± 1.43 ^ac^

Values are shown as mean ± SD of eight mice. NFD = Normal pellet diet; HFD = 45% Kcal high-fat diet; WL = Wasabi Leaf/Folium, *Wasabia japonica* (Miq.) Matsum extracts. ^a^ *p* < 0.01 and ^b^ *p* < 0.05—vs. intact control; ^c^ *p* < 0.01—vs. HFD control.

**Table 13:** Changes in the liver lipid peroxidation in NFD or HFD supplied mice.

Groups Items	Control		Reference	Test Materials—WL		
	Intact	HFD	Metformin	200 mg/kg	100 mg/kg	50 mg/kg
Malondialdehyde (nM/mg tissue)	9.46 ± 1.83	72.90 ± 16.60 ^a^	37.63 ± 14.29 ^ac^	20.63 ± 4.68 ^ac^	27.13 ± 10.38 ^ac^	35.45 ± 14.41 ^ac^

Values are shown as mean ± SD of eight mice. NFD = Normal pellet diet; HFD = 45% Kcal high-fat diet; WL = Wasabi Leaf/Folium, *Wasabia japonica* (Miq.) Matsum extracts. ^a^ *p* < 0.01 —vs. intact control; ^c^ *p* < 0.01—vs. HFD control.

**Table 14:** Changes in the antioxidant defense systems in NFD or HFD supplied mice.

Items Groups	Glutathione (μM/mg Tissue)	Catalase (U/mg Tissue)	SOD (U/mg Tissue)
Controls			
Intact	65.20 ± 13.43	66.10 ± 20.85	8.88 ± 1.79
HFD	8.83 ± 2.12 ^a^	10.60 ± 4.14 ^a^	0.83 ± 0.56 ^a^
Reference			
Metformin	31.32 ± 6.93 ^ac^	33.88 ± 10.15 ^ac^	3.50 ± 0.87 ^ac^
Test materials—WL			
200 mg/kg	56.87 ± 10.79 ^c^	51.89 ± 10.09 ^c^	6.48 ± 1.37 ^ac^
100 mg/kg	48.10 ± 13.94 ^bc^	44.00 ± 15.61 ^bc^	4.94 ± 1.39 ^ac^
50 mg/kg	34.03 ± 10.04 ^ac^	34.46 ± 13.53 ^ac^	3.59 ± 1.05 ^ac^

Values are shown as mean ± SD of eight mice. NFD = Normal pellet diet; HFD = 45% Kcal high-fat diet; WL = Wasabi Leaf/Folium, *Wasabia japonica* (Miq.) Matsum extracts; SOD = Superoxide dismutase. ^a^ *p* < 0.01 and ^b^ *p* < 0.05—vs. intact control; ^c^ *p* < 0.01—vs. HFD control.

**Table 15:** Changes in the hepatic glucose-regulating enzyme activities in NFD or HFD supplied mice.

Items Groups	Glucokinase (nM/min/mg Protein)	Glucose-6-Phosphatase (nM/min/mg Protein)	PEPCK (nM/min/mg Protein)
Controls			
Intact	5.12 ± 1.74	111.61 ± 25.62	1.56 ± 0.52
HFD	1.45 ± 0.52 ^a^	310.11 ± 52.77 ^a^	5.63 ± 0.83 ^a^
Reference			
Metformin	2.84 ± 0.63 ^ac^	170.43 ± 26.02 ^ac^	2.71 ± 0.80 ^ac^
Test materials—WL			
200 mg/kg	3.49 ± 0.77 ^bc^	145.71 ± 12.63 ^bc^	1.90 ± 0.29 ^c^
100 mg/kg	3.08 ± 0.53 ^ac^	152.83 ± 15.55 ^ac^	2.25 ± 0.38 ^bc^
50 mg/kg	2.90 ± 0.84 ^bc^	169.02 ± 27.58 ^ac^	2.65 ± 0.57 ^ac^

Values are shown as mean ± SD of eight mice. NFD = Normal pellet diet; HFD = 45% Kcal high-fat diet; WL = Wasabi Leaf/Folium, *Wasabia japonica* (Miq.) Matsum extracts; PEPCK = Phosphoenolpyruvate carboxykinase. ^a^ *p* < 0.01 and ^b^ *p* < 0.05—vs. intact control; ^c^ *p* < 0.01—vs. HFD control.

**Table 16:** Changes in lipid metabolism-related gene mRNA expressions in liver and adipose tissue of NFD or HFD supplied mice, realtime RT-PCR analysis.

Groups Items	Control		Reference	Test Materials—WL		
	Intact	HFD	Metformin	200 mg/kg	100 mg/kg	50 mg/kg
Hepatic tissue (Relative to control/GAPDH)						
ACC1	0.98 ± 0.08	5.22 ± 0.69 ^a^	2.58 ± 0.75 ^ab^	1.36 ± 0.19 ^ab^	1.89 ± 0.53 ^ab^	2.49 ± 0.76 ^ab^
AMPKα1	1.00 ± 0.07	0.25 ± 0.07 ^a^	0.57 ± 0.14 ^ab^	0.77 ± 0.17 ^ab^	0.66 ± 0.15 ^ab^	0.59 ± 0.19 ^ab^
AMPKα2	1.00 ± 0.08	0.22 ± 0.09 ^a^	0.52 ± 0.07 ^ab^	0.76 ± 0.12 ^ab^	0.66 ± 0.16 ^ab^	0.54 ± 0.17 ^ab^
Adipose tissue (Relative to control/GAPDH)						
Leptin	1.00 ± 0.08	8.04 ± 1.17 ^a^	3.19 ± 0.43 ^ab^	1.40 ± 0.34 ^ab^	2.11 ± 0.59 ^ab^	3.13 ± 1.25 ^ab^
UCP2	1.01 ± 0.08	0.22 ± 0.07 ^a^	0.54 ± 0.13 ^ab^	0.80 ± 0.10 ^ab^	0.71 ± 0.12 ^ab^	0.55 ± 0.14 ^ab^
Adiponectin	1.01 ± 0.07	0.15 ± 0.09 ^a^	0.47 ± 0.11 ^ab^	0.66 ± 0.16 ^ab^	0.59 ± 0.13 ^ab^	0.47 ± 0.13 ^ab^
C/EBPα	1.01 ± 0.03	2.84 ± 0.89 ^a^	1.62 ± 0.60 ^ab^	1.25 ± 0.11 ^ab^	1.46 ± 0.21 ^ab^	1.63 ± 0.30 ^ab^
C/EBPβ	1.00 ± 0.06	3.99 ± 0.69 ^a^	2.18 ± 0.78 ^ab^	1.34 ± 0.18 ^a^	1.97 ± 0.51 ^ab^	2.08 ± 0.46 ^ab^
SREBP1c	0.99 ± 0.05	2.72 ± 0.65 ^a^	1.62 ± 0.34 ^ab^	1.15 ± 0.10 ^ab^	1.36 ± 0.18 ^ab^	1.58 ± 0.32 ^ab^
PPARα	1.00 ± 0.07	0.17 ± 0.06 ^a^	0.45 ± 0.09 ^ab^	0.73 ± 0.14 ^ab^	0.62 ± 0.19 ^ab^	0.48 ± 0.13 ^ab^
PPARγ	1.00 ± 0.03	8.51 ± 0.59 ^a^	4.55 ± 1.06 ^ab^	2.33 ± 0.61 ^ab^	3.82 ± 1.37 ^ab^	4.41 ± 1.31 ^ab^
FAS	1.00 ± 0.07	14.92 ± 4.27 ^a^	5.09 ± 0.80 ^ab^	2.84 ± 0.82 ^ab^	3.65 ± 0.98 ^ab^	4.97 ± 1.25 ^ab^

Values are shown as mean ± SD of eight mice. NFD = Normal pellet diet; HFD = 45% Kcal high-fat diet; WL = Wasabi Leaf/Folium, *Wasabia japonica* (Miq.) Matsum extracts; GAPDH = Glyceraldehydes 3-phosphate dehydrogenase; ACC1 = Acetyl-CoA carboxylase 1; AMPK = 5’ adenosine monophosphate-activated protein kinase; UCP = Mitochondrial uncoupling protein; C/EBP = CCAAT-enhancer-binding protein; SREBP = Sterol regulatory element-binding protein; PPAR = Peroxisome proliferator-activated receptor; FAS = Fatty acid synthase. ^a^ *p* < 0.01—vs. intact control; ^b^ *p* < 0.01—vs. HFD control.
